# A New Projected Active Set Conjugate Gradient Approach for Taylor-Type Model Predictive Control: Application to Lower Limb Rehabilitation Robots With Passive and Active Rehabilitation

**DOI:** 10.3389/fnbot.2020.559048

**Published:** 2020-12-03

**Authors:** Tian Shi, Yantao Tian, Zhongbo Sun, Bangcheng Zhang, Zaixiang Pang, Junzhi Yu, Xin Zhang

**Affiliations:** ^1^College of Communication Engineering, Jilin University, Changchun, China; ^2^Department of Control Engineering, Changchun University of Technology, Changchun, China; ^3^Key Laboratory of Bionic Engineering of Ministry of Education, Jilin University, Changchun, China; ^4^School of Mechatronical Engineering, Changchun University of Technology, Changchun, China; ^5^State Key Laboratory for Turbulence and Complex Systems, Department of Advanced Manufacturing and Robotics, College of Engineering, Peking University, Beijing, China

**Keywords:** rehabilitation robot, model predictive control, intention recognition, conjugate gradient approach, projected operator

## Abstract

In this paper, a three-order Taylor-type numerical differentiation formula is firstly utilized to linearize and discretize constrained conditions of model predictive control (MPC), which can be generalized from lower limb rehabilitation robots. Meanwhile, a new numerical approach that projected an active set conjugate gradient approach is proposed, analyzed, and investigated to solve MPC. This numerical approach not only incorporates both the active set and conjugate gradient approach but also utilizes a projective operator, which can guarantee that the equality constraints are always satisfied. Furthermore, rigorous proof of feasibility and global convergence also shows that the proposed approach can effectively solve MPC with equality and bound constraints. Finally, an echo state network (ESN) is established in simulations to realize intention recognition for human–machine interactive control and active rehabilitation training of lower-limb rehabilitation robots; simulation results are also reported and analyzed to substantiate that ESN can accurately identify motion intention, and the projected active set conjugate gradient approach is feasible and effective for lower-limb rehabilitation robot of MPC with passive and active rehabilitation training. This approach also ensures computational when disturbed by uncertainties in system.

## 1. Introduction

The number of limb impairment patients who were injured by stroke has increased year by year, and this disease has also been developing in the direction of youth, seriously endangering the health of patients (Zorowitz et al., 2013). Recently, researchers have paid a great deal of attention to robotics to promote the development of scientific and engineering fields (Jin et al., 2017; Jin and Li, 2018; Xie et al., 2019, 2020; Zhang et al., 2020). Compared to traditional rehabilitation training methods that see problems like resource consumption, high costs, and long rehabilitation period, lower-limb rehabilitation robots can be deemed as a more effective method for recovering patients' movement function. In virtue of interaction generally existing between the lower-limb rehabilitation robot and the patient, to avoid the a second injury in the patient during rehabilitation training, it is essential to propose a human–machine interactive control method, which can be utilized to investigate and analyze the lower-limb rehabilitation robot (Fleischer and Hommel, 2008).

Intention recognition is one of the key points for realizing human–machine interactive control methods with lower-limb rehabilitation robots. Generally, motion intentions include joint angles and angular velocities, which can be recognized by decoding bioelectrical signals. Afterwards, the intentions are referenced by the lower-limb rehabilitation robot to complete interaction (Ding et al., 2016; Peng et al., 2018). An appropriate alternative is to establish a relationship between biological signals and movements for the patient. The surface electromyography (sEMG) can be regarded as the biological signals, which is a micro-electrical signal that appears 20–80 ms before the muscle contraction (Fleischer and Hommel, 2008). Involving two approaches to construct the relationship, one of which is a physiological muscle model, such as the Hill muscle model and the Hammerstein muscle model (Hunt et al., 1998; Buchanan et al., 2004), muscle forces and joint motions can be estimated by those models from sEMG signals; meanwhile, the unidentified physiological parameters of those models affect their applications in rehabilitation systems (Han et al., 2015). Another is the regression model, which can be established to connect sEMG signals to indicate intentions in a straightforward manner, rather than considering physiological parameters (Ding et al., 2016). For instance, a BP neural network was exploited to describe the relationship between sEMG signals and motion intentions, which was verified on able-bodied subjects and patients (Zhang et al., 2012); least squares support vector regression was proposed to predict periodic lower-limb motions from multi-channel sEMG signals (Li et al., 2015). Those approaches constitute a connection between human's bioelectrical signals and motion intentions, and intention recognition can thus be realized effectively.

During the rehabilitation training, the motion trajectory, which is a predetermined curve or recognized by sEMG signals, is known to the rehabilitation device, and the patient is assisted by the lower-limb rehabilitation robot to recover. However, it is very important to avoid the risk of second injury for patients in rehabilitation, and a human–machine interactive control method should therefore be considered to increase security and stability of rehabilitation robots. Recently, some classical control methods have been developed and applied to rehabilitation robots, for example, the rehabilitative system was realized by an adaptive control framework and a human–machine interactive method; meanwhile, the potential conflicts between patient and rehabilitation robot were rejected by position-dependent stiffness and predetermined trajectory (Zhang and Cheah, 2015). Pehlivan et al. (2016) presented a minimal assist-as-needed controller for rehabilitation robots, which could provide corresponding assistance for patients during rehabilitation training.

In recent years, model predictive control (MPC) not only considered the constraints of a non-linear system but predicted future states. People have therefore paid more attention to investigating it and applying it to the aerospace, automobile, economics, and robotics fields. As the patient should be assisted by the lower-limb rehabilitation robot within a relatively safe motion range and protected against accidents, the MPC method is suitable for human–machine interactive control of the lower-limb rehabilitation robot. Generally speaking, the MPC method is designed to optimize multivariable and constrained control systems. On the one hand, a control sequence is created by minimizing an optimization objective function over a finite prediction horizon within state and control constraints. On the other hand, the first optimal solution of non-linear optimization problem feeds back to the non-linear systems, which is utilized to generate the next iteration (Mayne, 2014). A key issue for MPC is that the computational burden of real-time optimization should be reduced through neural networks (Yan and Wang, 2014). To solve this problem is to linearize non-linear systems and discretize the differential term. More and more people have consequently developed some classical methods. For example, a neural network was utilized to identify the unknown non-linear discrete system, and then one-order Taylor expansion formula was used to linearize the MPC problem (Pan and Wang, 2012). For non-linear continuous systems, in order to discretize the differential term and guarantee the higher precision, a Taylor-type numerical differentiation formula was developed and applied to solve non-linear time-varying optimization problem (Zhang et al., 2019), non-linear time-varying equations (Jin et al., 2019), time-varying matrix inversion (Guo et al., 2017), future dynamic non-linear optimization problem (Wei et al., 2019), time-dependent Sylvester equations (Qi et al., 2020), and so on. As the MPC problem can be seen as an optimization problem, a Taylor-type numerical differentiation formula is thus also suitable for solving the MPC problem online.

Another key issue of MPC to be looked at further is online optimization. In fact, an MPC problem can be converted to a non-linear optimization problem with equality constraints and bound constraints and be solved to obtain the control sequence at each sample time. There are thus numerous algorithms proposed and studied for this non-linear optimization problem. A complex-valued discrete-time neural dynamics is studied by Qi et al. (2019) for solving time-dependent complex quadratic programming (QP), which possesses high accuracy and strong robustness but is only suitable for linear constrained QP problems. A trust region-sequential quadratic programming approach attempts to solve a sequence of QP subproblems of non-linear constraint optimization problems, which is based on trust-region technology and applied by finding the Karush-Kuhn-Tucker (KKT) points. It is noteworthy for the approach that the compatibility of the QP subproblem and the Maratos effect were overcome by adding several linear equations into the traditional trust region-SQP algorithm (Sun et al., 2019). However, the calculation of this algorithm is increased by the added linear equations. In addition, some troubles maybe emerge; for instance, one is the consistency of the coefficient matrix and the other the computational burden. Similarly, conjugate gradient methods can also be regarded as an effective optimization approach that utilizes an iteration point with a steep descent direction to generate conjugate direction and compute a global minimum point instead of solving linear equations of trust region-SQP algorithm (Sun et al., 2019). Some classical conjugate gradient methods include the Hestenes-Stiefel (HS) method (Hestenes and Stiefel, 1952), the Fletcher-Reeves (FR) method (Fletcher and Reeves, 1964), the Polak-Ribiére-Polyak (PRP) method (Polak and Ribière, 1969; Polyak, 1969), the Dai-Yuan (DY) method (Dai and Yuan, 2000a), the Liu-Storey (LS) method (Liu and Storey, 1991), and the conjugate descent (CD) method (Dai and Yuan, 2000b), but those methods were exploited to solve unconstrained optimization problems off-line. The MPC problem usually contains some constraint qualifications. Some modified conjugate gradient methods, which consider the constrained conditions, were thus proposed by modifying the search direction and a projected operator (Dai, 2014; Sun et al., 2018). Besides, a projected gradient method, which projected the gradient into the feasible region, was proposed by Rosen (1960), and some modified conjugate gradient methods were extended by some researchers based on the mentioned methods (Li and Li, 2013; Dai, 2014). Those modified conjugate gradient methods were also applied in optimal robust controllers and robots (Sun et al., 2018). In this paper, a modified conjugate gradient method, which will simultaneously consider equality constraints and bound constraints, will be utilized to solve MPC problem. Furthermore, the proposed algorithm of this paper is further applied to the lower limb rehabilitation robots with passive and active rehabilitation.

There are three significant contributions to be developed in this paper. The primary one is that a new projected active set conjugate gradient approach is developed and investigated, and rigorous proof of feasibility and global convergence is also given. The second is that a relationship between sEMG signals and motion intentions established by an echo state network (ESN) model can identify human motion state. Finally, a numerical simulation about passive and active rehabilitation training of lower-limb rehabilitation robot is illustrated and solved by the proposed method. Surprisingly, the studies on the rehabilitation training of lower-limb rehabilitation robot for MPC problem with projected active set conjugate gradient approach are scarce. This motivates our present study.

The rest of this paper is organized as follows: In section 2, the MPC problem is introduced, and a three-order Taylor-type numerical differentiation formula is proposed and utilized to discretize MPC model. In section 3, a new projected active set conjugate gradient algorithm is developed, analyzed, and investigated for the MPC problem, which can be generalized from non-linear systems and lower limb rehabilitation robots. Furthermore, the feasibility and the global convergence of this approach are also proven. The relationship of sEMG signals and motion intentions is established by an ESN model in section 5; meanwhile, passive and active rehabilitation training of lower-limb rehabilitation robot is illustrated and simulated by the proposed method, which is based on sEMG signals with ESN model and MPC problem. Furthermore, the disturbance of dynamic model is also considered through simulation in section 4. Finally, section 6 summarizes the results of lower-limb rehabilitation robot based on the MPC technique and expects future work.

## 2. From MPC to Non-linear Constrained Optimization

### 2.1. Problem Description

In this subsection, consider the following non-linear control system:

(1) $$\begin{array}{l} \{\begin{array}{c} {\dot{\text{x}}}^{k}=\text{A}\left({\text{x}}^{k}\right){\text{x}}^{k}+\text{B}\left({\text{x}}^{k}\right){\text{u}}^{k}+\text{C}\left({\text{x}}^{k}\right),\\ {\text{y}}^{k}=\text{h}\left({\text{x}}^{k}\right), \end{array} \end{array}$$

where **x**^*k*^ ∈ *R*^*n*^ is a system state variable, **u**^*k*^ ∈ *R*^*m*^ is a control input signal, **A**(**x**^*k*^) ∈ *R*^*n*×*n*^, **B**(**x**^*k*^) ∈ *R*^*n*×*m*^, **C**(**x**^*k*^) ∈ *R*^*n*^ are the state matrix, control input matrix and constant matrix, respectively. **y**^*k*^ ∈ *R*^*n*^ denotes the system output, and **h**(·) is a non-linear function.

MPC for the non-linear control system is devoted to generating a sequence of control signals by minimizing an objective function repeatedly over a finite moving prediction horizon with system state and input constraints satisfied simultaneously. For the non-linear control system (1), the MPC problem can be described as a non-linear discrete-time optimal control problem within input and state constraints:

(2) $$\begin{array}{l} \underset{{𝕏}^{k},{𝕌}^{k}}{\text{min}}\overset{N}{\underset{i=1}{\sum }}{\left\|\text{r}\left(k+i|k\right)-\text{y}\left(k+i|k\right)\right\|}_{Q}^{2}+\overset{N_{u}-1}{\underset{j=0}{\sum }}{\left\|\Delta \text{u}\left(k+j|k\right)\right\|}_{R}^{2}\\ \text{ s.t. }\dot{\text{x}}\left(k+i|k\right)=\text{A}\left(k+i|k\right)\text{x}\left(k+i|k\right)+\text{B}\left(k+i|k\right)\text{u}\left(k+j|k\right)\\ \;+\text{C}\left(k+i|k\right),\\ \;\text{y}\left(k+i|k\right)=\text{h}\left(\text{x}\left(k+i|k\right)\right),\\ \;\text{x}\left(k+i|k\right)\in \left[{\text{x}}_{\text{min}},{\text{x}}_{\text{max}}\right],\text{u}\left(k+j|k\right)\in \left[{\text{u}}_{\text{min}},{\text{u}}_{\text{max}}\right],\\ \;i=1,2,\ldots ,N,j=1,2,\ldots ,N_{u}, \end{array}$$

where **r**(*k* + *i*|*k*) and **y**(*k* + *i*|*k*) are the desired output and the predicted output for *i*th step ahead from *k*th sampling instant; Δ**u**(*k* + *j*|*k*) = **u**(*k* + *j*|*k*) − **u**(*k* + *j* − 1|*k*) denotes the control increment; *N* and *N*_*u*_ are the prediction horizon and control horizon, respectively; 𝕏^*k*^ = {**x**(*k* + 1|*k*), **x**(*k* + 2|*k*), …, **x**(*k* + *N*|*k*)}, ${𝕌}^{k}=\left\{\text{u}\left(k|k\right),\text{u}\left(k+1|k\right),\ldots ,\text{u}\left(k+N_{u}-1|k\right)\right\}$; *Q* ∈ *R*^*n*×*n*^ and *R* ∈ *R*^*m*×*m*^ are real positive definite matrix, ||·||_*Q*_ and ||·||_*R*_ are Euclidean norms defined as $\|x|{|}_{𝔄}=\sqrt{x^{T}𝔄x}$, where 𝔄 is a square matrix.

### 2.2. Three-Order Taylor-Type Discretization for MPC

In this subsection, a three-order Taylor-type numerical differentiation formula with truncation error of **O**(*h*^2^) is constructed for the first-order derivative approximation and is exploited to discretize MPC problem (Jin and Zhang, 2014). To obtain the higher-order truncation error, Guo et al. (2017) proposed a novel Taylor-type numerical differentiation formula for the time-varying matrix inversion.

**Lemma 1**. Assume that **x**^*k*^ ∈ *C*^4^[*a, b*] and **x**^*k*−4^, **x**^*k*−3^, **x**^*k*−2^, **x**^*k*−1^, **x**^*k*^, **x**^*k* + 1^ ∈ [*a, b*], *h* denotes the sampling gap. Subsequently, a three-order Taylor-type numerical differentiation formula can be obtained as follows:

(3) $$\begin{array}{l} {\dot{\text{x}}}^{k}\approx \frac{13{\text{x}}^{k+1}-7{\text{x}}^{k}+2{\text{x}}^{k-1}-10{\text{x}}^{k-2}+{\text{x}}^{k-3}+{\text{x}}^{k-4}}{24h} \end{array}$$

with a truncation error of **O**(*h*^3^).

**Proof**. See Guo et al. (2017).

According to the Lemma 1, the non-linear control system (1) can be discreted as

(4) $$\begin{array}{l} \{\begin{array}{c} {\text{x}}^{k+1}=\left(\frac{24}{13}h{\text{G}}^{k}+\frac{7}{13}I\right){\text{x}}^{k}-\frac{2}{13}{\text{x}}^{k-1}+\frac{10}{13}{\text{x}}^{k-2}-\frac{1}{13}{\text{x}}^{k-3}\\ \;-\frac{1}{13}{\text{x}}^{k-4}+\frac{24}{13}h{\text{B}}^{k}{\text{u}}^{k}+\frac{24}{13}h{\text{Z}}^{k}\\ {\text{y}}^{k}\;=\text{h}\left({\text{x}}^{k}\right), \end{array} \end{array}$$

where **f** = **A**(**x**)**x** + **B**(**x**)**u** + **C**(**x**), ${\text{G}}^{k}=\frac{\partial \text{f}}{\partial \text{x}}\left({\text{x}}^{k},{\text{u}}^{k}\right)$, ${\text{B}}^{k}=\text{B}\left({\text{x}}^{k}\right)=\frac{\partial \text{f}}{\partial \text{u}}\left({\text{x}}^{k},{\text{u}}^{k}\right)$, **Z**^*k*^ = **f**(**x**^*k*^, **u**^*k*^) − **G**^*k*^**x**^*k*^ − **B**^*k*^**u**^*k*^, *I* is an identity matrix.

Therefore, the MPC problem (2) can be rewritten in the following form:

(5) $$\begin{array}{l} \underset{{𝕏}^{k},{𝕌}^{k}}{\text{min}}\;\overset{N}{\underset{i=1}{\sum }}{\left\|\text{r}\left(k+i|k\right)-\text{y}\left(k+i|k\right)\right\|}_{Q}^{2}+\overset{N_{u}-1}{\underset{j=0}{\sum }}{\left\|\Delta \text{u}\left(k+j|k\right)\right\|}_{R}^{2}\\ \text{s.t. }{\text{x}}^{i+1}=\left(\frac{24}{13}h{\text{G}}^{i}+\frac{7}{13}I\right){\text{x}}^{i}-\frac{2}{13}{\text{x}}^{i-1}+\frac{10}{13}{\text{x}}^{i-2}-\frac{1}{13}{\text{x}}^{i-3}\\ \;-\frac{1}{13}{\text{x}}^{i-4}+\frac{24}{13}h{\text{B}}^{i}{\text{u}}^{j}+\frac{24}{13}h{\text{Z}}^{i},\\ \;{\text{y}}^{i}=\text{h}\left({\text{x}}^{i}\right),\\ \;{\text{x}}^{i}\in \left[{\text{x}}_{\text{min}},{\text{x}}_{\text{max}}\right],{\text{u}}^{j}\in \left[{\text{u}}_{\text{min}},{\text{u}}_{\text{max}}\right],\\ \;i=1,2,\ldots ,N,j=1,2,\ldots ,N_{u}, \end{array}$$

where **x**^*i*^ = **x**(*k* + *i*|*k*), **u**^*j*^ = **u**(*k* + *j*|*k*), and **y**^*i*^ = **y**(*k* + *i*|*k*), and another symbols see Appendix.

In general, the MPC problem can be solved by the conjugate gradient approach (Šantin and Havlena, 2011), and a sequence

$$\begin{array}{l} {\text{u}}^{\left(i\right)}:=\left[u_{0}^{\left(i\right)};u_{1}^{\left(i\right)};\ldots ;u_{N_{u}-1}^{\left(i\right)}\right] \end{array}$$

can be regarded as an initial control input sequence of the MPC problem at Step *i*. In addition, the first element $u_{0}^{*}$ of optimal solution u^*^ can be seen as a feedback control law for non-linear control system (1).

For simplicity, the MPC problem (5) is equivalent to the following non-linear optimization problem with linear equality constrain and bound constrain:

(6) $$\begin{array}{l} \underset{x}{\text{min}}\;\Gamma \left(x\right)\\ \text{s.t. }\Lambda x=b,\;x\in \Omega , \end{array}$$

where *x* = (**x**, **u**), Γ(·) is a continuously differentiable function, Λ is a constant matrix, *b* is a constant vector, and Ω = {*x* = (**x**, **u**)|**x**_min_ ≤ **x** ≤ **x**_max_, **u**_min_ ≤ **u** ≤ **u**_max_} is a bound constrained set.

## 3. Projected Active Set Conjugate Gradient Algorithm for Non-linear Constrained Optimization

In this section, a projected active set conjugate gradient algorithm is proposed to solve the following non-linear optimization problem:

(7) $$\begin{array}{l} \underset{x}{\text{min }}\Gamma \left(x\right)\\ \text{s.t. }\Lambda x=b,\\ \;x\in \Omega =\left\{x|s\leq x\leq t\right\}, \end{array}$$

and the convergence of the proposed approach is developed, investigated, and analyzed as follows.

### 3.1. Projected Active Set HS-Type Conjugate Gradient Algorithm

To further analyze projected active set conjugate gradient algorithm, some basic definitions and notions should be revisited in this subsection. Let x^*^ be a stationary point of (7), and consider the following active set:

$$\begin{array}{l} H^{*}=\left\{i:x_{i}^{*}=s_{i}\text{ or }x_{i}^{*}=t_{i}\right\}. \end{array}$$

Furthermore, define

$$\begin{array}{l} L^{*}=\left\{1,2,\ldots ,n\right\}\backslash H^{*}, \end{array}$$

as a set of free variables, where L^*^ is the complement of H^*^. Therefore, the KKT conditions for the problem (7) can be converted as follows:

$$\begin{array}{l} \{\begin{array}{cc} \left(s_{i}+t_{i}-2x_{i}^{*}\right)\nabla \Gamma_{i}\left(x^{*}\right)\geq 0, & \text{if }i\in H^{*},\\ \nabla \Gamma_{i}\left(x^{*}\right)=0, & \text{if }i\in L^{*}, \end{array} \end{array}$$

where ∇Γ_*i*_(*x*) is the *i*th element of the gradient for Γ at *x*. According to the literature (Kanzow and Klug, 2006), *H*(*x*) and *L*(*x*), which approximate the active set and the free variables set can be defined as follows:

$$\begin{array}{l} H\left(x\right)=\left\{i:s_{i}\leq x_{i}\leq s_{i}+\psi \left(x\right)\text{ or }t_{i}-\psi \left(x\right)\leq x_{i}\leq t_{i}\right\},\\ L\left(x\right)=\left\{i:s_{i}+\psi \left(x\right)<x_{i}<t_{i}-\psi \left(x\right)\right\}, \end{array}$$

where ψ(*x*) = min{ξ(*x*), ψ_0_} and $\xi \left(x\right)=\sqrt{\left\|x-P_{\Omega }\left(x-\nabla \Gamma \left(x\right)\right)\right\|}$, *P*_Ω_(·) is a projection function defined as *P*_Ω_(x) = argmin_ω ∈ Ω_||x − ω||_2_. ψ_0_ is a positive scalar, which should be sufficiently small. Furthermore, it should satisfy the following inequality:

$$\begin{array}{l} 0<\psi_{0}<\underset{i=1,2,\ldots ,n}{\text{min}}\frac{1}{3}\left(s_{i}-t_{i}\right). \end{array}$$

In what follows, let *x*^*k*^ be the point of iteration *k*, and for simplicity, we abbreviate *H*(*x*^*k*^) and *L*(*x*^*k*^) as *H*^*k*^ and *L*^*k*^. According to the literature (Cheng et al., 2014), the active set *H*^*k*^ will be divided into the following three parts:

(8) $$\begin{array}{l} H_{1}^{k}=\{i:x_{i}^{k}=s_{i}\text{ or }x_{i}^{k}=t_{i},\text{ and }(s_{i}+t_{i}-2x_{i}^{k})\nabla \Gamma_{i}^{k}\geq 0\},\\ H_{2}^{k}=\{i:s_{i}\leq x_{i}^{k}\leq s_{i}+\psi (x^{k})\text{ or }t_{i}-\psi (x^{k})\leq x_{i}^{k}\leq t_{i},\text{ and}\\ \;(s_{i}+t_{i}-2x_{i}^{k})\nabla \Gamma_{i}^{k}<0\},\\ H_{3}^{k}=\{i:s_{i}<x_{i}^{k}\leq s_{i}+\psi (x^{k})\text{ or }t_{i}-\psi (x^{k})\leq x_{i}^{k}<t_{i},\text{ and}\\ \;(s_{i}+t_{i}-2x_{i}^{k})\nabla \Gamma_{i}^{k}\geq 0\}. \end{array}$$

It is inferred that a search direction *d*^*k*^ can be constructed as a feasible direction of Γ at *x*^*k*^ if and only if $d_{i}^{k}\geq 0$, $i\in \left\{i:x_{i}^{k}=s_{i}\;\text{and}\;\nabla \Gamma_{i}^{k}\geq 0\right\}$ and $d_{i}^{k}\leq 0$, $i\in \left\{i:x_{i}^{k}=t_{i}\;\text{and}\;\nabla \Gamma_{i}^{k}\leq 0\right\}$.

It is demonstrated that the active set $H_{1}^{k}$ can be seen as the equality constraints of (7), and according to Rosen's gradient projection method (Rosen, 1960; Dai, 2014), an active set projection matrix is given as follows:

(9) $$\begin{array}{l} P^{k}=I^{k}-{\left(M^{k}\right)}^{T}{\left(M^{k}{\left(M^{k}\right)}^{T}\right)}^{-1}M^{k},\\ M^{k}=\left[\begin{array}{c} E^{k}\\ \Lambda \end{array}\right], \end{array}$$

where *E*^*k*^ satisfies $E_{\left(L,\cdot \right)}^{k}x^{k}=s_{L}$ and $E_{\left(U,\cdot \right)}^{k}x^{k}=-t_{U}$, $L=\left\{i:x_{i}^{k}=s_{i}\;\text{and}\;\left(s_{i}+t_{i}-2x_{i}^{k}\right)\nabla \Gamma_{i}^{k}\geq 0\right\}$, $U=\left\{i:x_{i}^{k}=t_{i}\;\text{and}\;\left(s_{i}+t_{i}-2x_{i}^{k}\right)\nabla \Gamma_{i}^{k}\geq 0\right\}$,

Hence the search direction *d*^*k*^ is defined by

(10) $$\begin{array}{l} d^{k}=\{\begin{array}{c} -P^{k}\nabla \Gamma^{k},\text{ if }k=0\text{ or }\exists i\in H_{1}^{k}\cup H_{2}^{k}\cup H_{3}^{k},\\ -P^{k}\nabla \Gamma^{k}+\beta_{k}^{HS}d^{k-1}-\zeta_{k}{\hat{z}}^{k-1},\text{ if }k\geq 1\text{ and }\forall i\in L^{k}, \end{array} \end{array}$$

where

$$\begin{array}{l} \beta_{k}^{HS}=\frac{{\left(P^{k}\nabla \Gamma^{k}\right)}^{T}z^{k-1}}{{\left(d^{k-1}\right)}^{T}v^{k-1}},\zeta_{k}=\frac{{\left(\nabla \Gamma^{k}\right)}^{T}d^{k-1}}{{\left(d^{k-1}\right)}^{T}v^{k-1}},\\ z^{k-1}=\nabla \Gamma^{k}-\nabla \Gamma^{k-1},{\hat{z}}^{k-1}=P^{k}z^{k-1},\\ v^{k-1}=z^{k-1}+\gamma w^{k-1},\gamma =\gamma_{0}+\text{max}\left\{0,-\frac{{\left(w^{k-1}\right)}^{T}z^{k-1}}{{\left(w^{k-1}\right)}^{T}w^{k-1}}\right\},\\ w^{k-1}=x^{k}-x^{k-1}, \end{array}$$

and γ_0_ is a positive constant. In what follows, the search direction *d*^*k*^ can be rigorously proved as a feasible descent direction of Γ at *x*^*k*^ for non-linear optimization problem (7).

**Theorem 1**. Suppose that *x*^*k*^ ∈ {*x*|Λ*x* = *b, x* ∈ Ω} holds, and *x*^*k*^ is not a stationary point of (7), *d*^*k*^ is defined by (10), then the search direction *d*^*k*^ is a feasible descent direction of Γ at *x*^*k*^ for non-linear optimization problem (7).

**Proof**. According to (8) and (10), and as per definition of the projection matrix, the following two cases can be generalized.

Case 1. If *k* = 0 or $\exists i\in H_{2}^{k}\cup H_{3}^{k}$, then the inequality can be directly computed as follows:

(11) $$\begin{array}{l} {\left(\nabla \Gamma^{k}\right)}^{T}d^{k}={\left(\nabla \Gamma^{k}\right)}^{T}\left(-P^{k}\nabla \Gamma^{k}\right)=-\|P\nabla \Gamma^{k}\|{\;}^{2}\leq 0. \end{array}$$

Case 2. If *k* ≥ 1 and ∀*i* ∈ *L*^*k*^, then the following inequality can be directly obtained:

(12) $$\begin{array}{l} {\left(\nabla \Gamma^{k}\right)}^{T}d^{k}={\left(\nabla \Gamma^{k}\right)}^{T}\left(-P^{k}\nabla \Gamma^{k}+\beta_{k}^{HS}d^{k-1}-\zeta_{k}{\hat{z}}^{k-1}\right)\\ \;=-\|P^{k}\nabla \Gamma^{k}\|{\;}^{2}+\frac{{\left(P^{k}\nabla \Gamma^{k}\right)}^{T}z^{k-1}}{{\left(d^{k-1}\right)}^{T}v^{k-1}}{\left(\nabla \Gamma^{k}\right)}^{T}d^{k-1}\\ \;-\frac{{\left(\nabla \Gamma^{k}\right)}^{T}d^{k-1}}{{\left(d^{k-1}\right)}^{T}v^{k-1}}{\left(\nabla \Gamma^{k}\right)}^{T}P^{k}z^{k-1}\\ \;=-\|P^{k}\nabla \Gamma^{k}\|{\;}^{2}+{\left(d^{k-1}\right)}^{T}\frac{\nabla \Gamma^{k}{\left(\nabla \Gamma^{k}\right)}^{T}P^{k}z^{k-1}}{{\left(d^{k-1}\right)}^{T}v^{k-1}}\\ \;-\frac{{\left(d^{k-1}\right)}^{T}\nabla \Gamma^{k}}{{\left(d^{k-1}\right)}^{T}v^{k-1}}{\left(\nabla \Gamma^{k}\right)}^{T}P^{k}z^{k-1}\\ \;=-\|P^{k}\nabla \Gamma^{k}\|{\;}^{2}\leq 0. \end{array}$$

The search direction *d*^*k*^ is therefore a descent direction of Γ at *x*^*k*^.

Now, the proof of the feasibility for *d*^*k*^ is shown as follows, and it is further inferred that

$$\begin{array}{l} M^{k}\left(P^{k}\nabla \Gamma^{k}\right)=M^{k}\left(I-{\left(M^{k}\right)}^{T}{\left(M^{k}{\left(M^{k}\right)}^{T}\right)}^{-1}M^{k}\right)\nabla \Gamma^{k}=0. \end{array}$$

If *E*^*k*^ is not an empty set, it can be seen that

(13) $$\begin{array}{l} M^{k}d^{k}=M^{k}\left(-P^{k}\nabla \Gamma^{k}\right)=0. \end{array}$$

If *E*^*k*^ is an empty set, then *M*^*k*^ = Λ. Owing to Equation (10), then the following equation can be generalized as

(14) $$\begin{array}{l} M^{k}d^{k}=M^{k}\left(-P\nabla \Gamma^{k}+\beta_{k}^{HS}d^{k-1}-\zeta_{k}{\hat{z}}^{k-1}\right)\\ \;=-\Lambda P\nabla \Gamma^{k}+\beta_{k}^{HS}\Lambda d^{k-1}-\zeta_{k}\Lambda P\left(\nabla \Gamma^{k}-\nabla \Gamma^{k-1}\right)\\ \;=\beta_{k}^{HS}\Lambda d^{k-1}. \end{array}$$

If *k* = 0 or $\exists i\in H_{1}^{k}\cup H_{2}^{k}\cup H_{3}^{k}$, then

(15) $$\begin{array}{l} \Lambda d^{k}=-\Lambda P^{k}\nabla \Gamma^{k}=0. \end{array}$$

Owing to Equations (13)–(15), then we have *M*^*k*^*d*^*k*^ = 0 for all *k* ≥ 0. It is also inferred that *d*^*k*^ is a feasible descent direction for non-linear optimization problem (7).      □

According to the above analysis and investigation, the projected active set HS-type conjugate gradient algorithm (PASHS) is developed, analyzed, and verified for non-linear optimization problem (7).

**Algorithm 1. (PASHS)**

Step 0. Initialize *x*^0^ ∈ {*x*|Λ*x* = *b, x* ∈ Ω} and projection matrix P^0^, let *k* = 0 and positive constants ε, ψ_0_, γ_0_, η_0_, δ, ρ < 1.

Step 1. If $\left\|P_{\Omega }\left(x^{k}-P^{k}\nabla \Gamma^{k}\right)-x^{k}\right\|\leq \varepsilon$ or *k* > *k*_max_, stop; else go to Step 2.

Step 2. Compute *d*^*k*^ by (10).

Step 3. Determine a stepsize $\eta_{k}=\text{max}\left\{\eta_{0}\rho^{j}|j=0,1,2,\ldots \right\}$ by Armijio-type line search rule:

(16) $$\begin{array}{l} \Gamma \left(x^{k}+\eta_{k}d^{k}\right)\leq \Gamma \left(x^{k}\right)+\delta \eta_{k}{\left(P^{k}\nabla \Gamma^{k}\right)}^{T}d^{k}. \end{array}$$

Step 4. Let $x^{k+1}=P_{\Omega }\left(x^{k}+\eta_{k}d^{k}\right)$, and *k*: = *k* + 1, go to Step 1.

**Remark 1**. A projected matrix P^*k*^ is computed by an active set, which ensures iteration point satisfying equality and bounded constraints of non-linear optimization problem (7). Assume that if the component the active set *H*^*k*^ is not contained in the previous iteration point *x*^*k*^, the search direction *d*^*k*^ is updated by the second formula of (10); otherwise, the search direction *d*^*k*^ is generated by the projected gradient method, which is the first formula of (10). Furthermore, combining with Armijio-type line search, it can be proved that the proposed PASHS algorithm guarantees the feasibility and global convergence for non-linear optimization problem (7).

### 3.2. Convergence Analysis

In this subsection, to further investigate the convergence of the Algorithm 1 (PASHS) for non-linear optimization problem (7), some basic assumptions should be revisited and introduced in this subsection.

**Assumption 1**. The level set

(17) $$\begin{array}{l} D=\left\{x\in \Omega |\Gamma \left(x\right)\leq \Gamma \left(x^{0}\right),\Lambda x=b\right\} \end{array}$$

is bounded.

**Assumption 2**. Given that the objective function Γ:*R*^*n*^ → *R* is continuously differentiable on an open set *N* ⊆ *D* and its gradient is Lipschitz continuous, there exists a positive constant *W* > 0 that satisfies the following inequality:

(18) $$\begin{array}{l} \|\nabla \Gamma \left(x\right)-\nabla \Gamma \left(y\right)\|\leq W\|x-y\|,\;\forall x,y\in N. \end{array}$$

As {Γ(*x*^*k*^)} is a descending sequence, it is clear that the sequence {*x*^*k*^} generated by Algorithm 1 (PASHS) is contained in *D*. In addition, according to Assumption 1, it is inferred that the gradient of Γ is bounded, i.e., there exists a positive constant γ > 0 such that

(19) $$\begin{array}{l} \|\nabla \Gamma \left(x\right)\|\leq \gamma ,\;\forall x\in D. \end{array}$$

Since the matrix P is a projected matrix, suppose that there exists a positive constant *C* > 0, and the following inequality can be obtained:

(20) $$\begin{array}{l} \|P\nabla \Gamma \left(x\right)\|\leq C,\;\forall x\in D. \end{array}$$

**Lemma 2**. Assume that the iterative sequence {*x*^*k*^} generated by Algorithm 1 (PASHS). The step size η^*k*^ is obtained via the Armijo line search rule (16), and then there exists a positive constant *c*_0_ > 0 such that the following inequality holds

(21) $$\begin{array}{l} \eta_{k}\geq c_{0}\frac{\|P^{k}\nabla \Gamma^{k}\|{\;}^{2}}{\|d^{k}\|{\;}^{2}} \end{array}$$

for sufficiently large *k*.

**Proof**. According to Armijio-type linear search rule (16), the following inequality can be obtained:

(22) $$\begin{array}{l} \overset{\infty }{\underset{i=1}{\sum }}-\delta \eta_{k}{\left(P^{k}\nabla \Gamma^{k}\right)}^{T}d^{k}\leq \Gamma \left(x^{0}\right)-\Gamma \left(x^{*}\right)<+\infty . \end{array}$$

Combined (11) and (12), and the properties of the projected matrix P^*k*^, the inequality can be generalized as follows

(23) $$\begin{array}{l} \overset{\infty }{\underset{i=1}{\sum }}\eta_{k}\|P^{k}\nabla \Gamma^{k}\|{\;}^{2}=\overset{\infty }{\underset{i=1}{\sum }}\eta_{k}{\left(\nabla \Gamma^{k}\right)}^{T}P^{k}\nabla \Gamma^{k}\\ \begin{array}{l} \;=-\overset{\infty }{\underset{i=1}{\sum }}\eta_{k}{\left(P^{k}\nabla \Gamma^{k}\right)}^{T}d^{k}<+\infty . \end{array} \end{array}$$

Now, the following two cases can be taken into account and be utilized to prove (21).

Case 1: If η_*k*_ = 1, according to Equations (11), (12), and (23), and by applying the Cauchy-Schwarz inequality, we can derive that

$$\begin{array}{l} \|P^{k}\nabla \Gamma^{k}\|{\;}^{2}=|{\left(P^{k}\nabla \Gamma^{k}\right)}^{T}d^{k}|\leq \|P^{k}\nabla \Gamma^{k}\|\cdot \|d^{k}\|. \end{array}$$

Thus, the inequality (21) holds.

Case 2: If η_*k*_ < 1, assume that the Armijio-type line search rule is not true, there thus exists a positive constant $\rho^{-1}\eta_{k}$ such that the following inequality holds true:

(24) $$\begin{array}{l} \Gamma \left(x^{k}+\rho^{-1}\eta_{k}d^{k}\right)-\Gamma \left(x^{k}\right)>\delta \rho^{-1}\eta_{k}{\left(P^{k}\nabla \Gamma^{k}\right)}^{T}d^{k}. \end{array}$$

Using the mean-value theorem and Assumption 1, there exists a positive constant ξ_*k*_ ∈ (0, 1) such that $x^{k}+\xi_{k}\rho^{-1}\eta_{k}d^{k}\in D$ and

(25) $$\begin{array}{l} \Gamma (x^{k}+\rho^{-1}\eta_{k}d^{k})-\Gamma (x^{k})=\rho^{-1}\eta_{k}\nabla \Gamma {\left(x^{k}+\xi_{k}\rho^{-1}\eta_{k}d^{k}\right)}^{T}d^{k}\\ \;=\rho^{-1}\eta_{k}{\left(\nabla \Gamma^{k}\right)}^{T}d^{k}+\rho^{-1}\eta_{k}(\nabla \Gamma (x^{k}\\ \;+\xi_{k}\rho^{-1}\eta_{k}d^{k})-\nabla \Gamma^{k}{)}^{T}d^{k}\\ \;\leq \rho^{-1}\eta_{k}{\left(\nabla \Gamma^{k}\right)}^{T}d^{k}+W\rho^{-2}{\left(\eta_{k}\right)}^{2}\|d^{k}{\|}^{2}. \end{array}$$

Combining inequality (24) and Theorem 1, the following inequality can be directly computed as

(26) $$\begin{array}{l} \eta_{k}\geq \frac{\left(1-\delta \right)\rho }{W}\frac{\|P^{k}\nabla \Gamma^{k}\|{\;}^{2}}{\|d^{k}\|{\;}^{2}}. \end{array}$$

Let *c*_0_ = min{1, (1 − δ)ρ/*W*}, the conclusion is true.      □

**Lemma 3**. Suppose that Assumption 2 holds. The iterative sequence {*x*^*k*^} is generated by Algorithm 1 (PASHS), and then the search direction *d*^*k*^ defined by (10) is bounded, in other words, there exists a positive constant *M* ≥ 0 such that

(27) $$\begin{array}{l} \|d^{k}\|\leq M,\forall k\in {\mathbb{N}}^{*}. \end{array}$$

**Proof**. According to the Assumption 2 and Algorithm 1 (PASHS), the following inequality can be directly obtained:

$$\begin{array}{l} {\left(d^{k-1}\right)}^{T}v^{k-1}>\gamma_{0}\eta_{k-1}\|d^{k-1}\|{\;}^{2}. \end{array}$$

In addition, in term of the search direction (10) and Theorem 1, the following inequality can be derived as

(28) $$\begin{array}{l} \left\|d^{k}\|\leq \|P^{k}\nabla \Gamma^{k}\|+|\beta_{k}^{HS}|\cdot \|d^{k-1}\|+|\zeta_{k}|\cdot \|P^{k}z^{k-1}\right\|\\ \;\leq \|P\nabla \Gamma^{k}\|+\frac{\left\|{\left(P^{k}\nabla \Gamma^{k}\right)}^{T}z^{k-1}\right\|}{\gamma_{0}\eta_{k-1}\|d^{k-1}\|{\;}^{2}}\|d^{k-1}\|\\ \;+\frac{\left\|{\left(P^{k}\nabla \Gamma^{k}\right)}^{T}d^{k-1}\right\|}{\gamma_{0}\eta_{k-1}\|d^{k-1}\|{\;}^{2}}\|P^{k}z^{k-1}\|\\ \;\leq \|P^{k}\nabla \Gamma^{k}\|+\frac{\left\|\left(P^{k}\nabla \Gamma^{k}\right)\right\|}{\gamma_{0}\eta_{k-1}\|d^{k-1}\|}\left(\left\|z^{k-1}\|+\|P^{k}z^{k-1}\right\|\right)\\ \;\leq \|P^{k}\nabla \Gamma^{k}\|+\frac{\|\left(P^{k}\nabla \Gamma^{k}\right)\|W\left(1+\lambda_{\text{max}}\left(P^{k}\right)\right)}{\gamma_{0}}\\ \;\leq C\left(1+\frac{W\left(1+\lambda_{\text{max}}\left(P^{k}\right)\right)}{\gamma_{0}}\right). \end{array}$$

where $\lambda_{\text{max}}\left(P^{k}\right)$ is the maximum eigenvalue of the projected matrix P^*k*^.      □

**Theorem 2**. Suppose that Assumption 1 holds. The iterative sequence {*x*^*k*^} is generated by Algorithm 1 (PASHS), and then

(29) $$\begin{array}{l} \underset{k\to \infty }{\text{lim}}\text{inf}\|P^{k}\nabla \Gamma^{k}\|=0. \end{array}$$

**Proof**. According to (21), there exists a positive constant *c*_0_ such that

(30) $$\begin{array}{l} \eta_{k}\|d^{k}\|{\;}^{2}\geq c_{0}\|P^{k}\nabla \Gamma^{k}\|{\;}^{2}. \end{array}$$

Combining Algorithm 1 with Lemma 3, it implies that

$$\begin{array}{l} \underset{k\to \infty }{\text{lim}}\eta_{k}\|d^{k}\|{\;}^{2}=0. \end{array}$$

The following inequality can be generalized as *k* → ∞,

(31) $$\begin{array}{l} 0=\underset{k\to \infty }{\text{lim}}\frac{\eta_{k}}{c_{0}}\|d^{k}\|{\;}^{2}\geq \underset{k\to \infty }{\text{lim}}\text{inf}\|P^{k}\nabla \Gamma_{k}\|{\;}^{2}\geq 0. \end{array}$$

Hence ${\text{lim}}_{k\to \infty }\text{inf}\|P^{k}\nabla \Gamma^{k}\|=0$.      □

**Remark**. Owing to Theorem 2, it can be seen that the Algorithm 1 (PASHS) is globally convergent for the non-linear optimization problem (7). Combing the ESN learning algorithm and Algorithm 1 (PASHS), the optimal controller of the MPC problem can thus be solved rapidly; this is used for the patients through a lower-limb rehabilitation robot with passive and active rehabilitation training.

## 4. Simulations and Results

In this section, the proposed PASHS algorithm with MPC technique is applied to the passive rehabilitation training of the two-link lower-limb rehabilitation robot. Moreover, combining the ESN model and intention recognition, the MPC and PASHS algorithm also are utilized to active rehabilitation training.

### 4.1. Two-Link Lower-Limb Rehabilitation Robot With MPC

**Figure 1:**
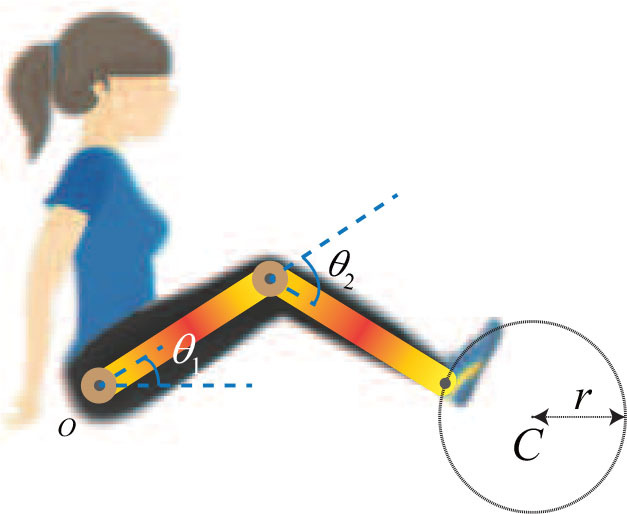
The schematic of two-link lower-limb rehabilitation robot.

The general dynamic model of two-link lower-limb rehabilitation robot is shown as follows (He et al., 2015):

(32) $$\begin{array}{l} \text{D}\left(\text{q}\right)\ddot{\text{q}}+\text{C}\left(\text{q},\dot{\text{q}}\right)\dot{\text{q}}+\text{G}\left(\text{q}\right)=\tau , \end{array}$$

where **q**, $\dot{\text{q}}$, $\ddot{\text{q}}\in R^{2}$ are angle, angular velocity and angular acceleration of hip and knee, respectively; **τ** ∈ *R*^2^ is a torque for the rehabilitation robot, which represents admissible control inputs; **D**(**q**) ∈ *R*^2×2^ is a positive-definite inertia matrix; $\text{C}\left(\text{q},\dot{\text{q}}\right)\in R^{2\times 2}$ is a centrifugal and Coriolis term; and **G**(**q**) ∈ *R*^2^ is related to gravity term. The state space expression of (32) can be described as

(33) $$\begin{array}{l} \{\begin{array}{c} \left[\begin{array}{c} \dot{\text{q}}\\ \ddot{\text{q}} \end{array}\right]=\left[\begin{array}{cc} 0 & I_{2\times 2}\\ 0 & -{\text{D}}^{-1}\left(\text{q}\right)\text{C}\left(\text{q},\dot{\text{q}}\right) \end{array}\right]\left[\begin{array}{c} \text{q}\\ \dot{\text{q}} \end{array}\right]+\left[\begin{array}{c} 0\\ {\text{D}}^{-1}\left(\text{q}\right) \end{array}\right]\tau \\ \;-\left[\begin{array}{c} 0\\ {\text{D}}^{-1}\left(\text{q}\right)\text{G}\left(\text{q}\right) \end{array}\right],\\ \text{y}\;=\text{h}\left(\text{q}\right), \end{array} \end{array}$$

where **y** ∈ *R*^2^ is the end-effector position coordinates, **h**(·) is a function mapping angles of the rehabilitation robot to the position coordinates. The schematic of a two-link lower-limb rehabilitation robot is shown in Figure 1.

As shown in **Figure 3**, θ_1_ = *q*_1_, θ_2_ = *q*_2_, *C* and *r* represent the the hip joint angle, the knee joint angle, center, and radius of the reference trajectory (which can be defined as a circle), respectively. The lengths of the links are *l*_1_ = 0.35 m and *l*_2_ = 0.32 m; the mass and inertia of two links are *m*_1_ = 1.8 kg, *m*_2_ = 1.65 kg and $I_{1}=\frac{1}{4}m_{1}l_{1}^{2}$ kg·m, $I_{2}=\frac{1}{4}m_{2}l_{2}^{2}$ kg·m, respectively; the gravity constant is *g* = 9.801 m/s^2^. In addition, the center and radius are *C* = (0.5, 0) and *r* = 0.1 m, respectively.

The parameters of the algorithm 1 (PASHS) are chosen as follows:

$$\begin{array}{l} \varepsilon =10^{-6},\;\psi_{0}=10^{-5},\;\gamma_{0}=10^{-3},\;\delta =0.0001,\;\rho =0.5,\;M^{0}=\Lambda , \end{array}$$

and the initial step size is selected as (Dai, 2011)

$$\begin{array}{l} \eta_{0}=\left|\frac{-\gamma_{0}\nabla \Gamma^{kT}d^{k}}{d^{kT}\left(\nabla \Gamma \left(x^{k}+\gamma_{0}d^{k}\right)-\nabla \Gamma^{k}\right)}\right|. \end{array}$$

Due to the proposed MPC model in section 3, the parameters of the MPC will be defined as follows:

$$\begin{array}{l} Q=R=I_{2\times 2}, \end{array}$$

the prediction horizon is *N* = 5 and the control horizon is *N*_*u*_ = 5; the sampling time is *h* = 0.01s. The initial state of end-effector position coordinates is *y*_0_ = (0.6, 0), and the motion-task duration is 25 s with 5 s per cycle. The following experiments are conducted under different constraints of torque: while the hip joint angle and the angular velocity are constrained in interval $\left[-\frac{2}{3}\pi ,\frac{2}{3}\pi \right]$, and [−π, π], the knee joint angle and the angular velocity are constrained in interval $\left[-\frac{4}{3}\pi ,0\right]$, and [−π, π] (Jin and Zhang, 2011).

### 4.2. The Passive Rehabilitation Training With Different Torques Constraints

**Figure 2:**
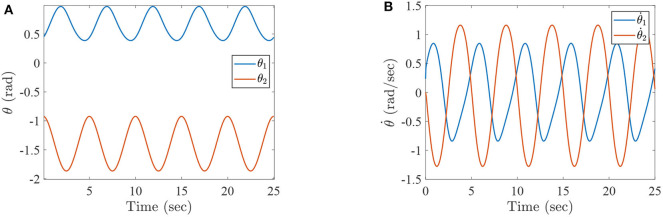
The simulation results with passive rehabilitation training, **(A)** the angles of lower-limb rehabilitation robot with constraints (34), and **(B)** the angular velocities of lower-limb rehabilitation robot with constraints (34).

**Figure 3:**
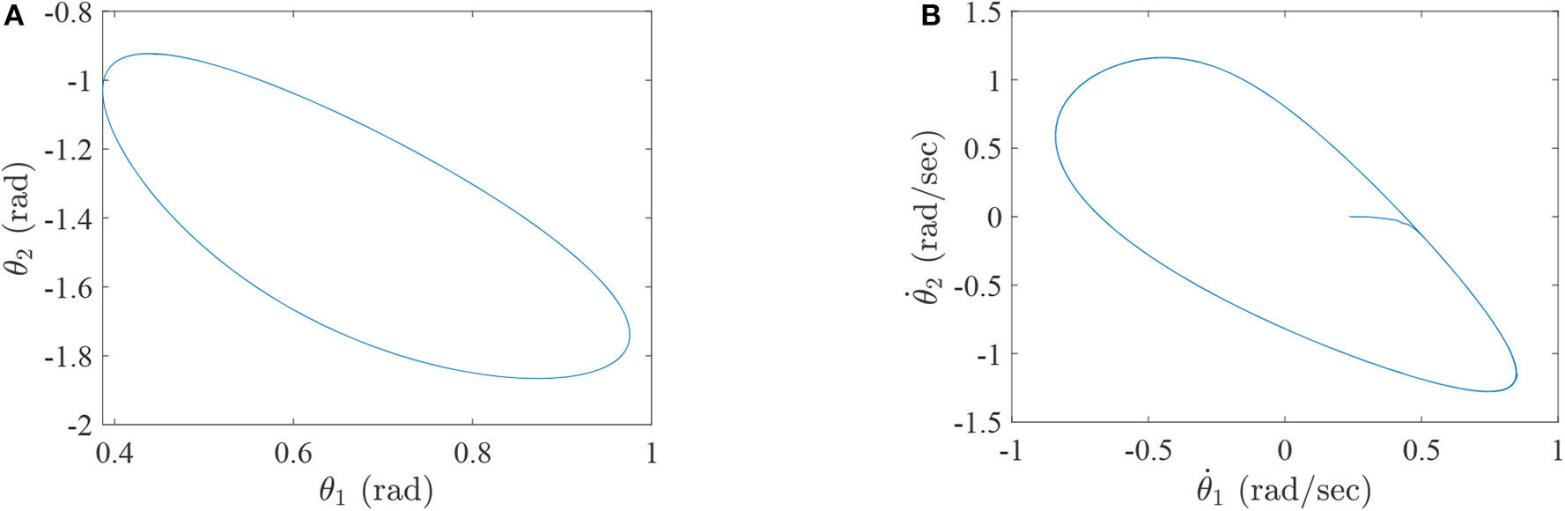
The simulation results with passive rehabilitation training, **(A)** the phase portraits for angles of lower-limb rehabilitation robot with constraints (34), **(B)** the phase portraits for angular velocities of lower-limb rehabilitation robot with constraints (34).

**Figure 4:**
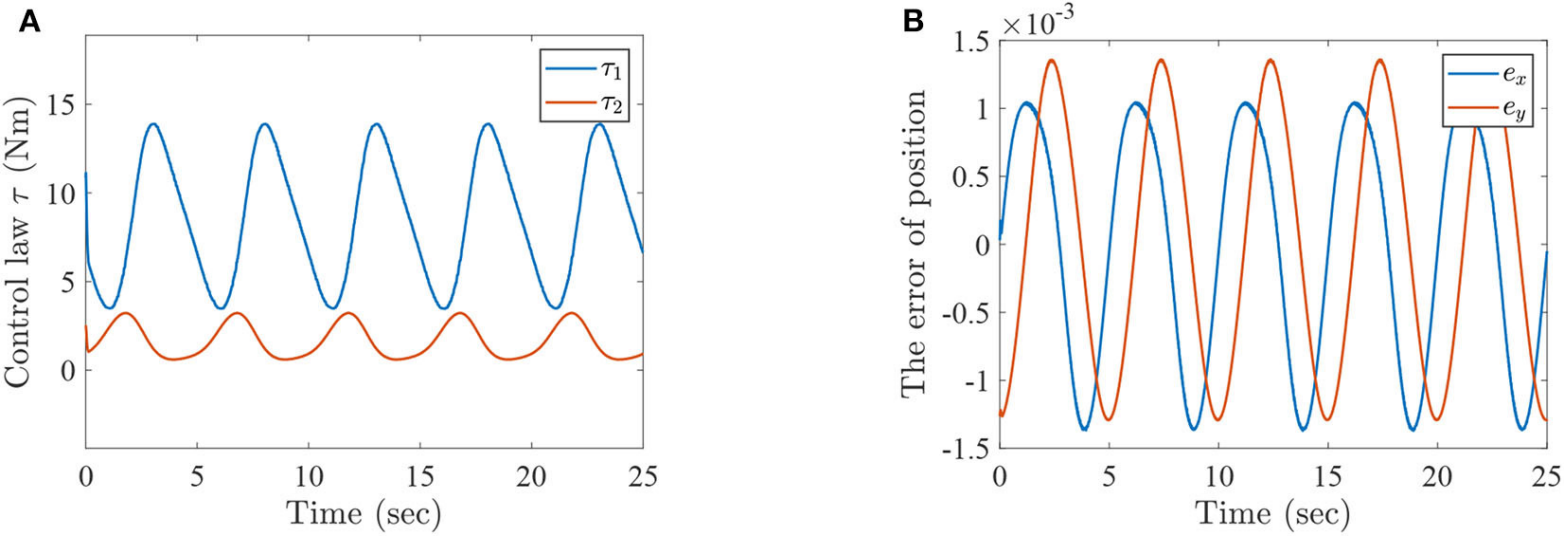
The simulation results with passive rehabilitation training, **(A)** the torques of lower-limb rehabilitation robot with constraints (34), and **(B)** the tracking errors of lower-limb rehabilitation robot with constraints (34).

**Example 1**: Consider the following control torques constraints

(34) $$\begin{array}{l} -15\text{ N}\cdot \text{ m}\leq \tau_{1},\tau_{2}\leq 15\text{ N}\cdot \text{ m}, \end{array}$$

where τ_1_ and τ_2_ correspond to the torques of the hip joint and the knee joint of a two-link lower-limb rehabilitation robot, respectively. The numerical results of this situation are shown in Figures 2–4.

Figure 2 represents the curves of angle and angular velocities of the hip and knee for the two-link lower-limb rehabilitation robot, and Figure 3 is the limit cycles of angle and angular velocities. From Figures 2, 3, it is further inferred that the angles and angular velocities of the lower-limb rehabilitation robot present the periodic properties; it also verifies that the proposed approach is feasible and effective. Figure 4A denotes the control torque vs. time, and the hip joint and knee joint of the rehabilitation robot can be controlled by torques τ_1_ and τ_2_, respectively. As shown in Figure 4A, it can be seen that the control torques change periodically for two-link lower-limb rehabilitation robot, which can help the injured patients to do rehabilitation training stably via Algorithm 1 (PASHS) with MPC technique. Figure 4B represents the tracking errors of the real position of end-effector and desired trajectory, while *e*_*x*_ and *e*_*y*_ are the tracking errors of horizontal ordinate and longitudinal coordinates. As shown in Figure 4B, the absolute value of the tracking errors is also smaller than 0.002 m, which also infers that the lower-limb rehabilitation robot could implement the passive rehabilitation training efficiently by the desired trajectory and MPC technique. It thus further demonstrates that the theoretical analyses are feasible and reliable. Besides, it is very important to take into consideration the energy consumption of rehabilitation training in real-world rehabilitation implementations, therefore, control torques should be constrained within relatively reasonable bounds. As shown in Figure 4A, the optimal control input is obtained by online solving of the MPC problem via Algorithm 1 (PASHS). However, it can be seen from Figure 4A, that the bounded condition is too large for non-linear optimization problem with MPC model, that is, the real-time control torque τ_1_ is around 13 N·m. In other words, to achieve the low-energy consumption, the boundary constraints can be reduced to around 10 N·m, which are utilized to control the two-link lower limb rehabilitation robot to realize the rehabilitation cycle movement of the injured lower limb.

**Example 2**: Consider the following control torques with constraint conditions

(35) $$\begin{array}{l} -10\text{ N}\cdot \text{ m}\leq \tau_{1},\tau_{2}\leq 10\text{ N}\cdot \text{ m}. \end{array}$$

**Figure 5:**
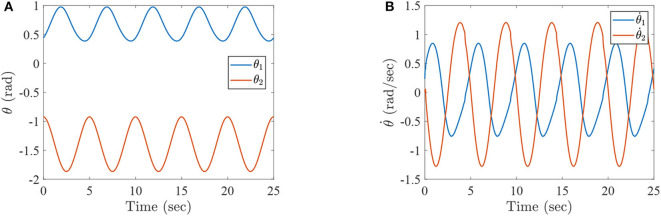
The simulation results with passive rehabilitation training, **(A)** the angles of lower-limb rehabilitation robot with constraints (35), and **(B)** the angular velocities of lower-limb rehabilitation robot with constraints (35).

**Figure 6:**
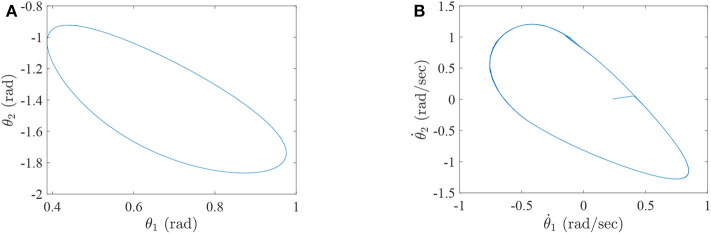
The simulation results with passive rehabilitation training, **(A)** the phase portraits for angles of lower-limb rehabilitation robot with constraints (35), and **(B)** the phase portraits for angular velocities of lower-limb rehabilitation robot with constraints (35).

**Figure 7:**
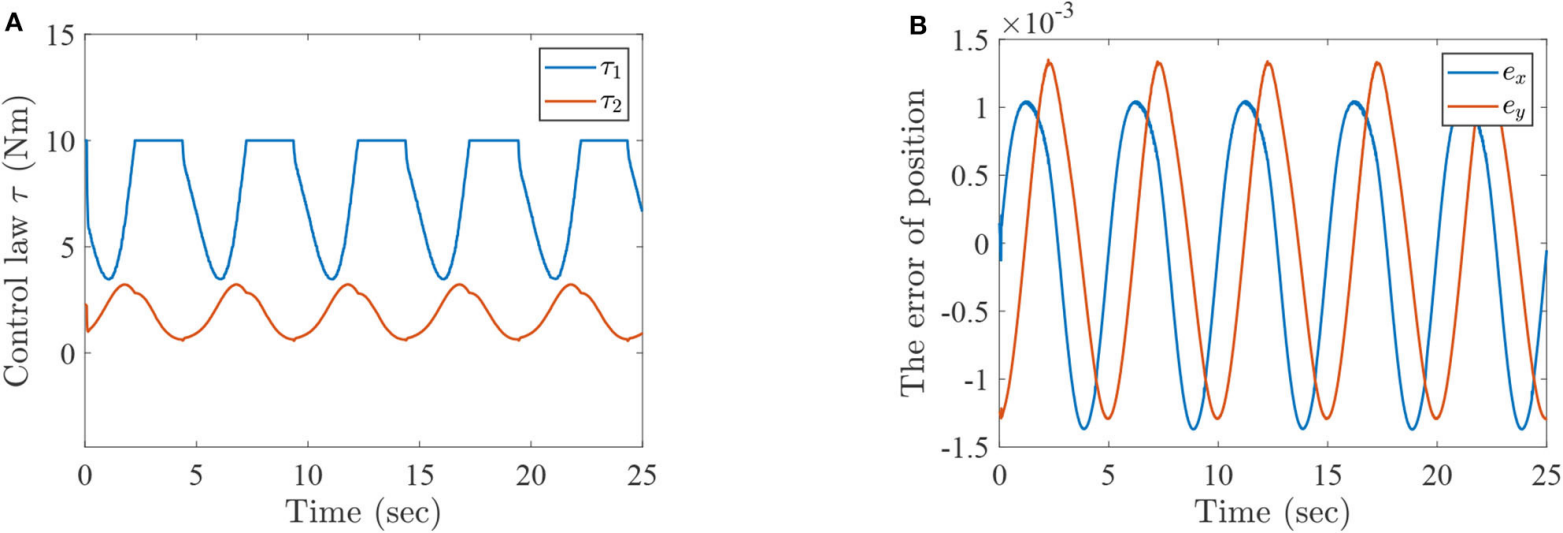
The simulation results with passive rehabilitation training, **(A)** the torques of lower-limb rehabilitation robot with constraints (35), and **(B)** the tracking errors of lower-limb rehabilitation robot with constraints (35).

The numerical results of this situation are shown in Figures 5–7.

Figure 5 represents the curves of angle and angular velocities for the two-link lower-limb rehabilitation robot, and Figure 6 shows the limit cycles of angle and angular velocities, respectively. As can be seen from Figures 5, 6, the angular velocities of the lower limb rehabilitation robot are affected by the control torques with constraint conditions limited to −10–10 N·m. However, the injured limb can stably complete rehabilitation training activities via the algorithm 1 (PASHS) with MPC technique. Figure 7A shows the control torque vs. time, and it can be seen that the control input τ_1_ could be constrained in between −10 and 10 N·m, and the smoothness of angular velocities maybe influenced by the constraint conditions. However, it further infers that the stability can be maintained for the two-link lower limb rehabilitation robot. Figure 7B plots the tracking errors of the real trajectories of end-effector and desired trajectories, while *e*_*x*_ and *e*_*y*_ are the same with the definition of Example 1. The proposed approach is therefore suitable for passive rehabilitation training of lower-limb rehabilitation robot.

**Example 3**: This example shows a comparison of different algorithms with the MPC solution.

**Figure 8:**
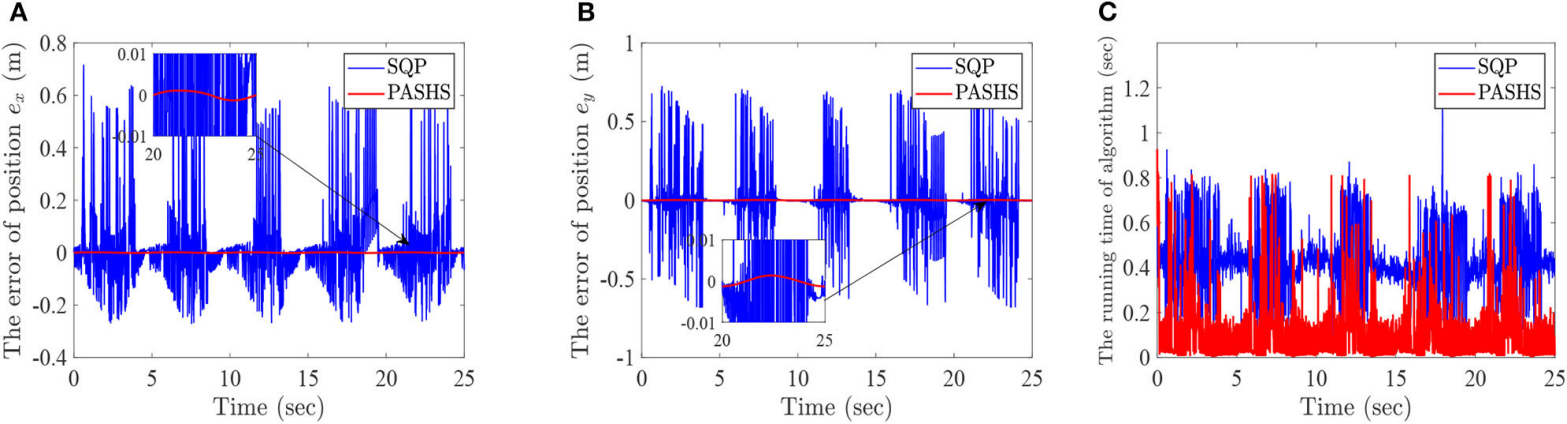
The simulation results with PASHS and SQP, **(A)** the tracking errors of horizontal ordinate, **(B)** the tracking errors of longitudinal coordinates, and **(C)** the running time of algorithm at every time.

In order to compare the advantages of PASHS algorithm, sequential quadratic programming (SQP) is selected to compare with our algorithm (Sun et al., 2020). The simulation problem is chosen as example 1, and the results are as follows:

Figure 8A represents the horizontal ordinate tracking errors *e*_*x*_ of lower-limb rehabilitation robots with passive rehabilitation training, and Figure 8B means the longitudinal ordinate tracking errors *e*_*y*_. From those two figures, it can be seen that the tracking errors of SQP are almost > 0.1 m at every iteration, and sometimes the errors were closed to 1m. This is because an optimal solution for SQP may not be in a feasible region. However, the tracking errors of PASHS are always smaller than 0.01 at every time. The optimal solution of the PASHS algorithm was satisfied by the constraint conditions because of the projective matrix and active set. PASHS was therefore more suitable for the MPC of lower-limb rehabilitation robots. Figure 8C is the running time of two algorithms at every optimization; according to this figure, the running time of PASHS was nearly always smaller than 0.2 s, and the SQP running time was around of 0.4 s. This can account for the real-time computing capability of PASHS algorithm with the MPC technique.

**Example 4**: The example with parameters' perturbation 1.5 times.

This example is reported the influence of uncertainties in system, and the 1.5 times parameters' perturbation is introduced into lower-limb rehabilitation robots. During the simulation, the parameters are selected as *l*_1_ = 0.35 m, *l*_2_ = 0.32 m, *m*_1_ = 2.7 kg, *m*_2_ = 2.475 kg, *I*_1_ = 0.0827 kg·m, and *I*_2_ = 0.0634 kg·m. Other conditions are the same as Example 1. The results of this example are shown as follows.

**Figure 9:**
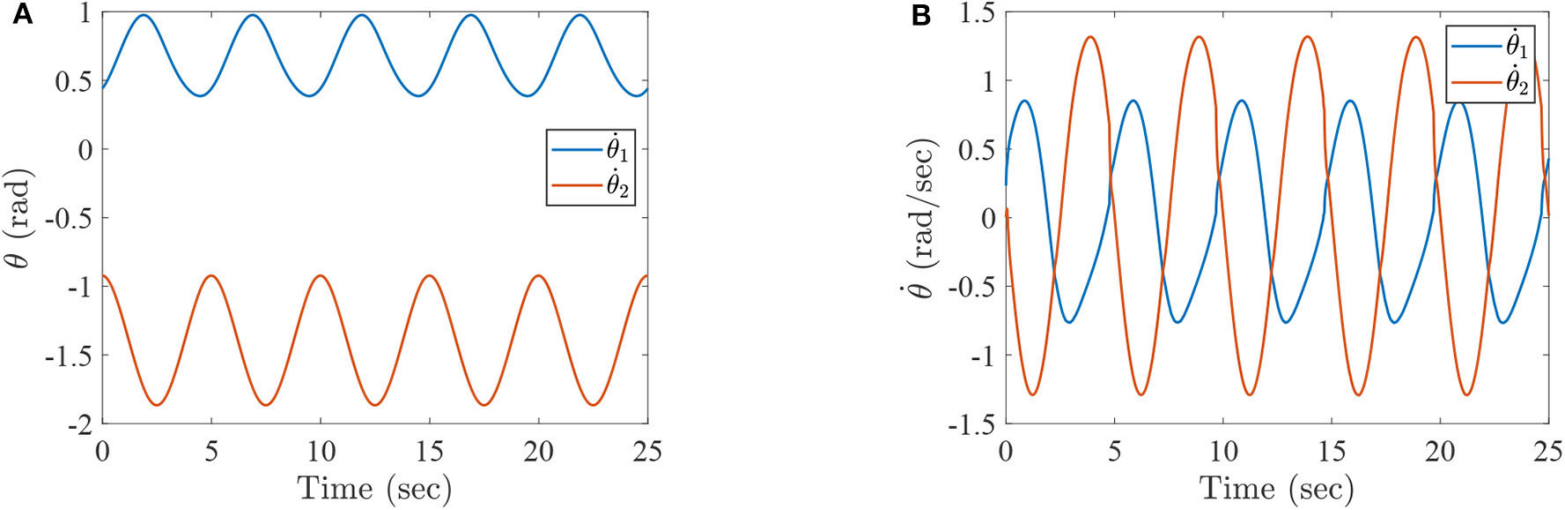
The simulation results with 1.5 times parameters' perturbation, **(A)** the angles of lower-limb rehabilitation robot with constraints (34), and **(B)** the angular velocities of lower-limb rehabilitation robot with constraints (34).

**Figure 10:**
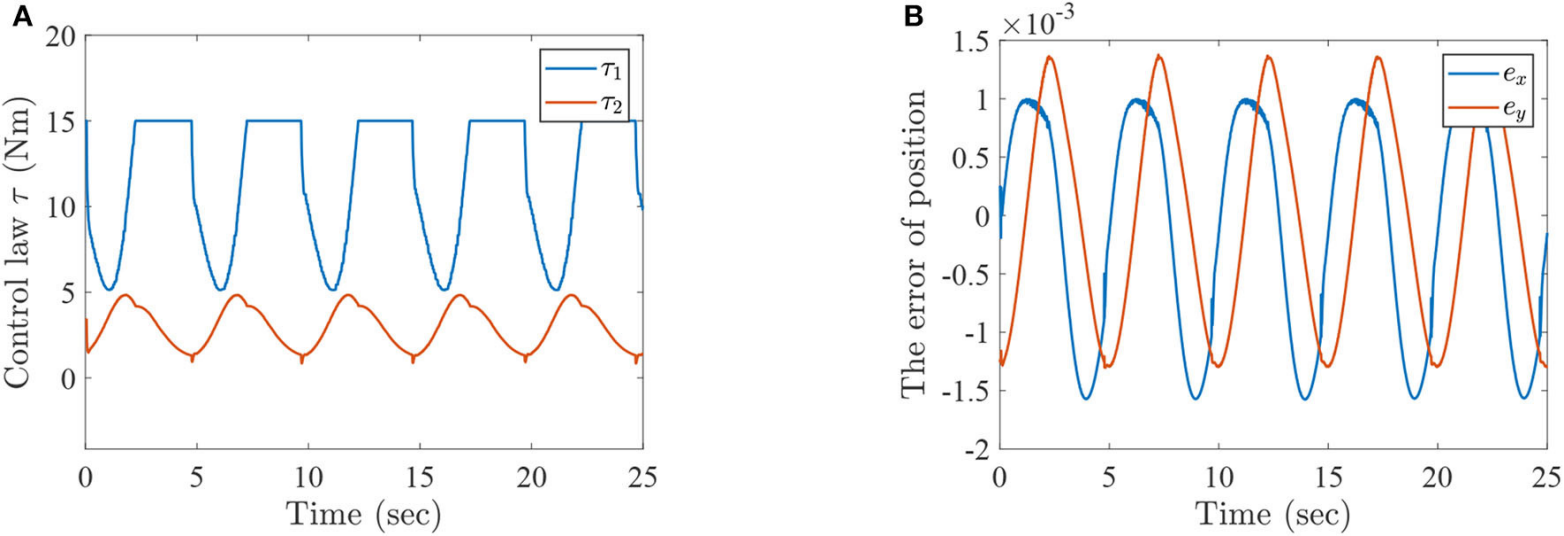
The simulation results with 1.5 times parameters' perturbation, **(A)** the torques of lower-limb rehabilitation robot with constraints (34), and **(B)** the tracking errors of lower-limb rehabilitation robot with constraints (34).

Figures 9A,B represent the angles and angular velocities of lower-limb rehabilitation robot with 1.5 times parameters' perturbation, respectively. As can be seen from these figures, the angles and angular velocities are changed periodically and stably, although the model is disturbed. Figure 10A is the torques of lower-limb rehabilitation robot which is subjected to 1.5 times parameter perturbation, and Figure 10B represents the horizontal ordinate tracking errors *e*_*x*_ and the longitudinal ordinate tracking errors *e*_*y*_ of lower-limb rehabilitation robots with 1.5 times parameters' perturbation. From Figure 10A, we can find that torques are limited between −15 and 15 N·m because the mass of lower-limb rehabilitation robot is added. However, the absolute value of tracking errors are smaller than 0.0015 m according to Figure 10B. The PASHS algorithm could thus solve MPC problems of the lower-limb rehabilitation robot with uncertainties in the model, and high accuracy could also be guaranteed.

### 4.3. sEMG-Based Active Rehabilitation Training

**Figure 11:**
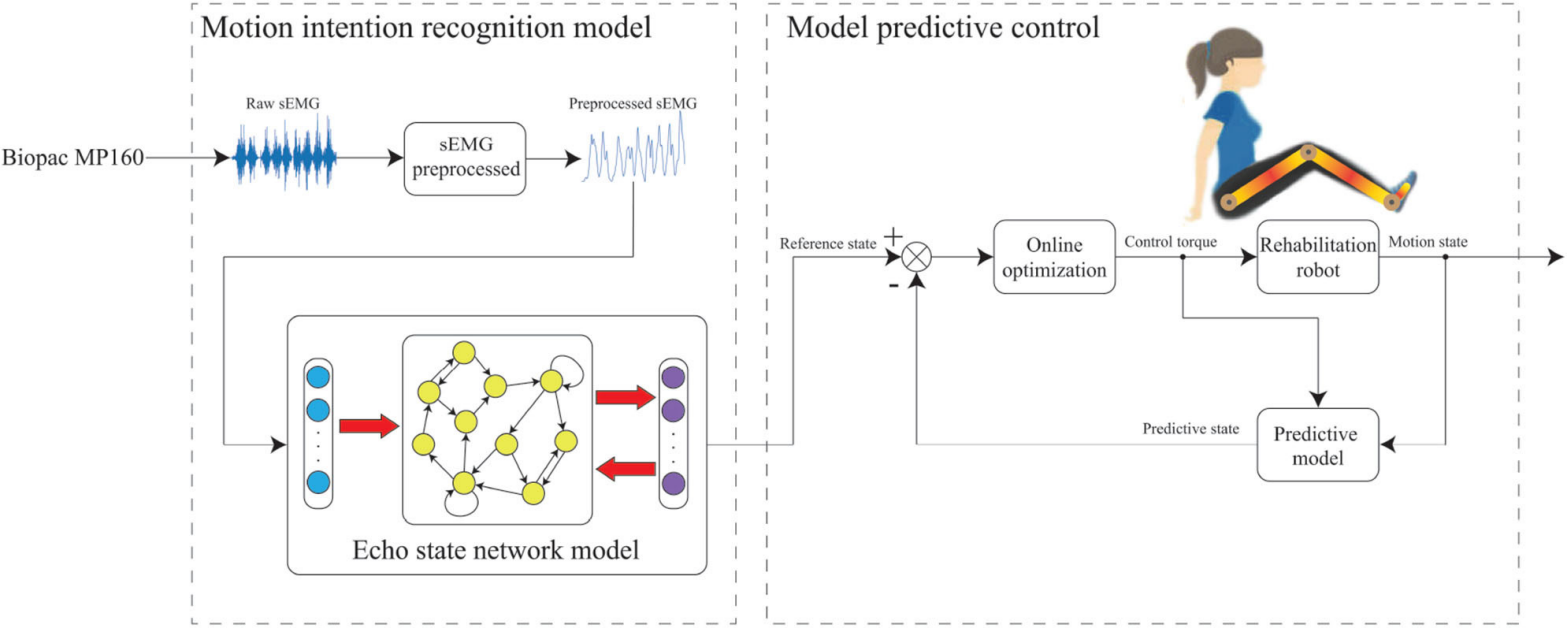
The flowsheet of intention recognition and rehabilitation training.

In this subsection, the active intention of injured patients is regarded as one of the most important rehabilitation steps. Furthermore, the joint trajectories of injured lower limb can be identified via the mentioned ESN model based on the active motion intention, which can be seen as the desired trajectories of lower limb rehabilitation robots. A numerical simulation is illustrated and analyzed for two-link lower limb rehabilitation robot with ESN model and MPC technique. The technical diagram of sEMG-based active rehabilitation training and intention recognition is shown in Figure 11.

The active rehabilitation training consists of two parts. The first one is intention recognition, which collects and preprocesses raw sEMG signals, and then motion intention is identified by the ESN model. The details of first part is described in the following description.

**Figure 12:**
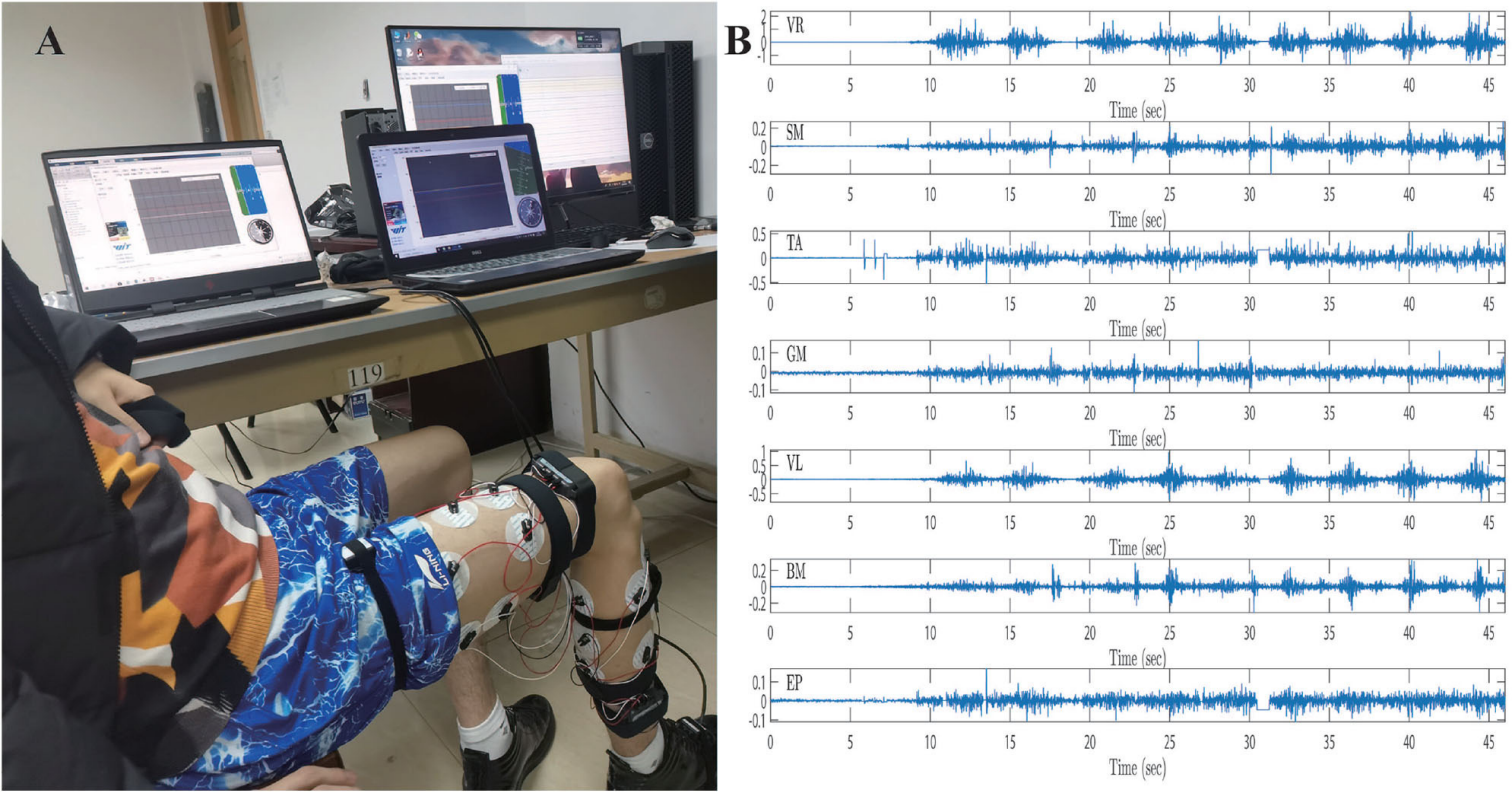
The intention recognition experiment, **(A)** the sEMG signals sampling of the experiment procedure, **(B)** the raw sEMG signals of subject.

During the data acquisition stage, a subject sits on a chair and swings the shank periodically. The sEMG signals of seven muscles of leg, which include the vastus rectus muscle (VR), semitendinosus muscle (SM), tibialis anterior muscle (TA), gastrocnemius muscle (GM), vastus lateralis muscle (VL), biceps muscle of thigh (BM), and extensor pollicis longus (EP), need to be recorded through data acquisition unit, respectively (Tong et al., 2014). The acquisition device is BIOPAC MP160, which can simultaneously capture eight channels of sEMG signals at the default 2 kHz sample rate. Angles and angular velocities of knee and ankle are recorded by inertial measurement unit (IMU), which selects 100 Hz as the sample rate. Due to the sample rate of sEMG signals is higher than IMU, the sub-sampling technology should be implemented by (Zhang et al., 2012)

$$\begin{array}{l} {\text{sEMG}}_{\text{pre}}\left(k\right)=\text{sEMG}\left(20k-19\right), \end{array}$$

where sEMG_pre_(*k*) represents the sEMG signals after sub-sampling at k times. The position of the electrodes and raw sEMG signals are shown in Figure 12.

We then use neural network technology to establish the relationship between sEMG signals and motion state. This is due to the original sEMG signals being contaminated by different measurement noises, such as direct current bias and baseline noise (Law et al., 2011). The raw sEMG signals need to be preprocessed, which includes a high-pass filter with 50 Hz high cut-off frequency, full-wave rectification technology, low-pass filter with 5 Hz low cut-off frequency and normalized technology (Han et al., 2015). The sEMG signals can be seen as the input signals of neural network when the noise of raw sEMG signals is eliminated by the mentioned methods.

**Figure 13:**
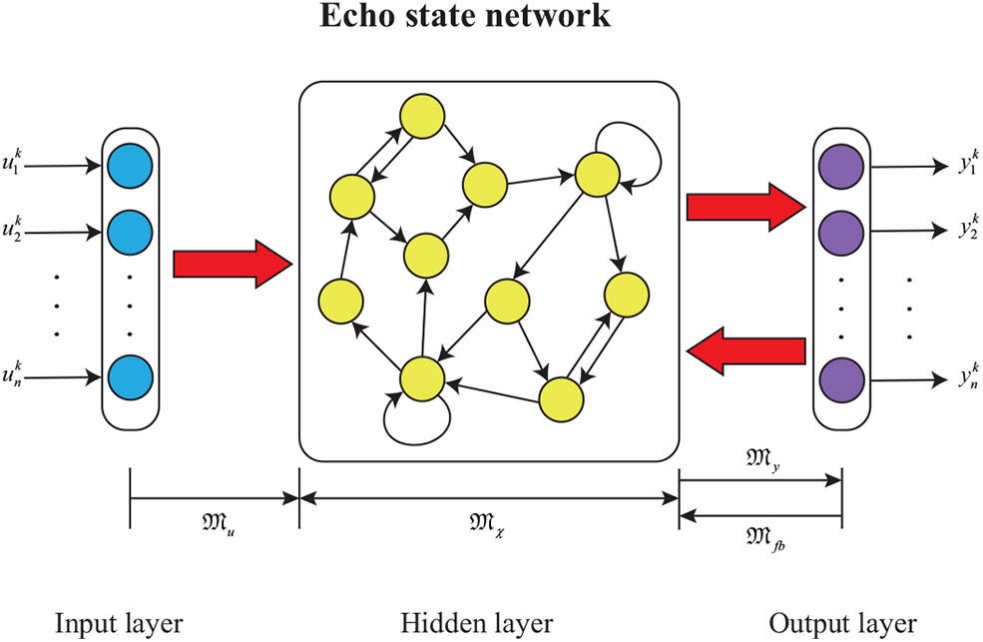
The ESN diagram.

The ESN model is a kind of recurrent neural network that is composed of an input layer, a hidden layer where neurons interconnect randomly, and an output layer. The network architecture is depicted in Figure 13.

The mathematical model of the ESN model can be obtained as

(36) $$\begin{array}{l} X^{k+1}=F\left({𝔐}_{X}X^{k}+{𝔐}_{U}U^{k+1}+{𝔐}_{F}Y^{k}\right),\\ Y^{k+1}={𝔐}_{Y}X^{k+1}, \end{array}$$

where F(·) is an activation function and commonly generates from F(x)=tanh(x); ${𝔐}_{X}\in R^{l\times l}$, ${𝔐}_{U}\in R^{l\times n}$, ${𝔐}_{F}\in R^{l\times m}$, ${𝔐}_{Y}\in R^{m\times l}$ are the internal connection weight of the hidden layer, the input layer to the hidden layer connection weight matrix, the output layer to the hidden layer feedback weight matrix, and the hidden layer to the output layer connection weight matrix; X and Y are the echo state and output vectors of the ESN model, respectively. Assume that ${𝔐}_{X}$, ${𝔐}_{U}$, and ${𝔐}_{F}$ are unmodifiable during the ESN model training (Pan and Wang, 2012). The ESN algorithm with off-line learning is summarized as follows.

**Algorithm 2. (ESN learning algorithm)**

Step 0. Initialize echo state $X^{0}$ and randomly obtain a matrix 𝔐. Normalize $\hat{𝔐}=𝔐/|\lambda_{\text{max}}|$ and generate ${𝔐}_{X}=\alpha_{X}\hat{𝔐}$, ${𝔐}_{U}\in R^{l\times n}$, ${𝔐}_{F}\in R^{l\times m}$, where $\alpha_{X}<1$ is a spectral radiuses of ${𝔐}_{X}$ and |λ_max_| is a spectral radius of 𝔐.

Step 1. Compute the echo state by

$$\begin{array}{l} X^{k+1}=F\left({𝔐}_{X}X^{k}+{𝔐}_{U}{\hat{U}}^{k+1}+{𝔐}_{F}{\hat{Y}}^{k}\right) \end{array}$$

for *k* = 0, 1, …, *N*, where ${\hat{U}}^{k}$ and ${\hat{Y}}^{k}$ are the *k*th input and output reference data from the training dataset.

Step 2. Collect the reservoir state **X** and target state **Y** as follows:

$$\begin{array}{l} \text{X}=\left[X^{1},\ldots ,X^{N}\right],\text{Y}=\left[{\hat{Y}}^{1},\ldots ,{\hat{Y}}^{N}\right]. \end{array}$$

Step 3. Off-line compute matrix ${𝔐}_{Y}={\left({\text{X}}^{+}\text{Y}\right)}^{T}$, where **X**^+^ is the pseudo-inverse of **X**.

**Figure 14:**
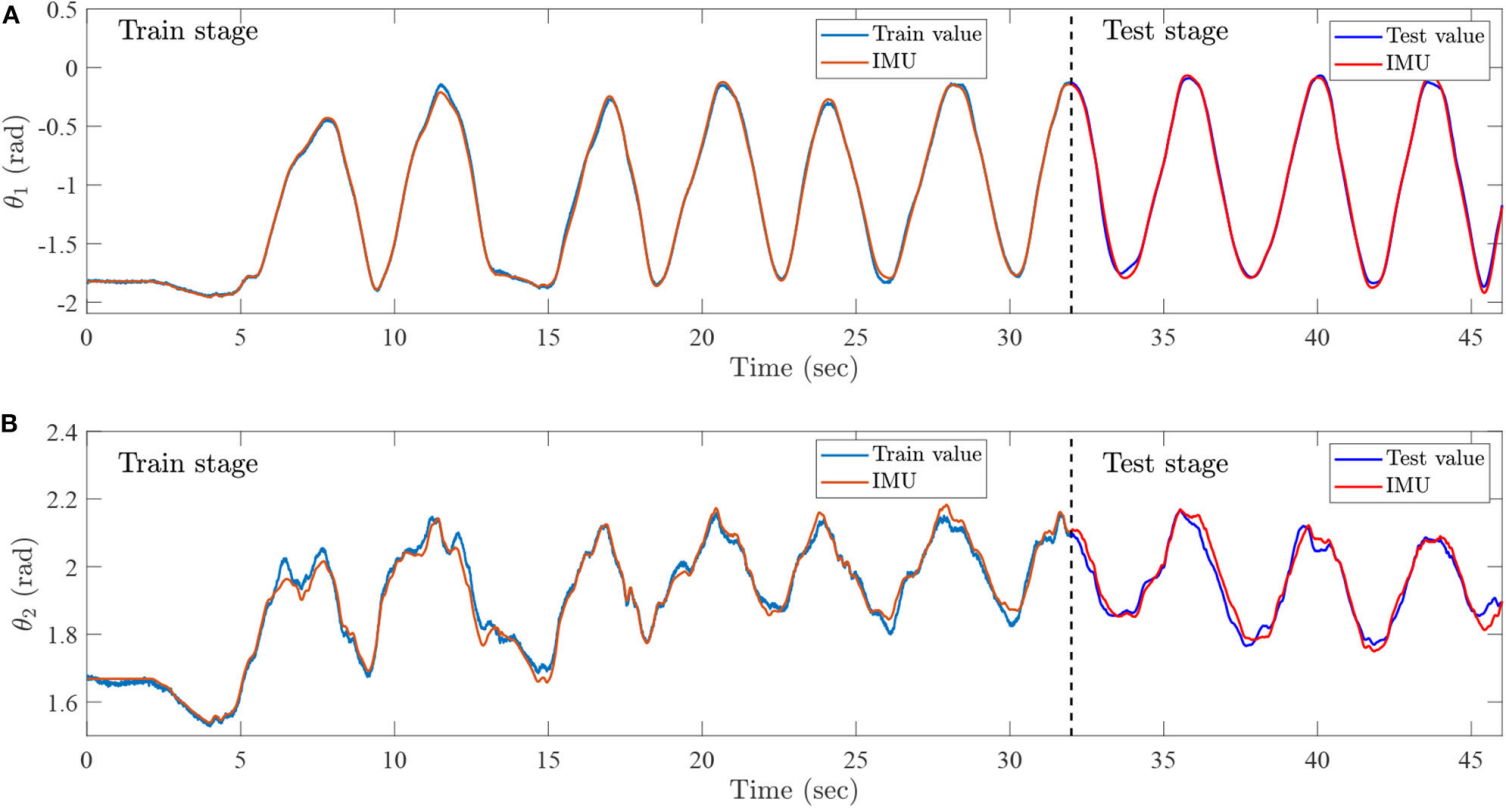
Intention recognition results for angles, **(A)** the knee angle, which is recognized by the sEMG and ESN model, and **(B)** the ankle angle, which is recognized by sEMG and ESN model.

**Figure 15:**
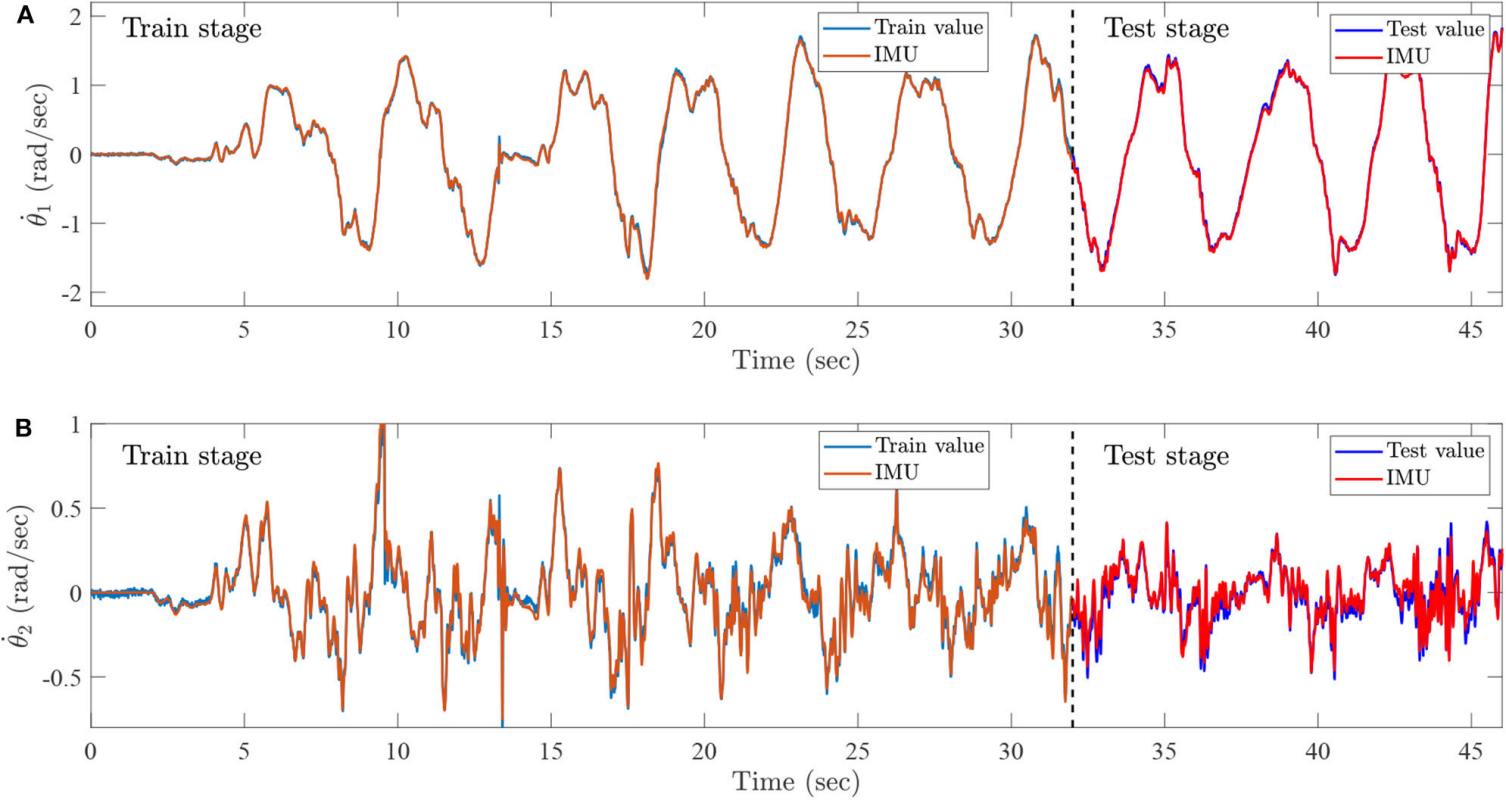
Intention recognition results for angular velocities, **(A)** the knee angular velocity, which is recognized by sEMG and ESN model, and **(B)** the ankle angular velocity, which is recognized by the sEMG and ESN model.

During the training process, the preprocessed sEMG signals are recorded by the Biopac MP160 system with seven channels from 0 to 46 s. Furthermore, the joint trajectories of injured lower limb are recorded by IMU, the data set from 0 to 32 s is then collected as a training set, and the remainder of the data set is regarded as a test set. The training set is utilized to train the ESN model, and the testing set is exploited to simulate lower limb rehabilitation robots with active rehabilitation training. The real angles and angular velocities are recorded by IMU, which aims at demonstrating the accuracy of proposed method. The ESN model has seven input neurons and four output neurons, the numbers of ESN hidden layer neurons are 100, and the parameters are selected as follows: $\alpha_{X}=0.5$. The results of ESN training and testing are shown in Figures 14, 15.

Figure 14 represents the intention recognition results of injured lower limb via ESN model, where θ_1_ and θ_2_ mean knee and ankle angles, respectively. Red solid lines denote real joint trajectories of knee and ankle angles, and blue solid lines represent training and testing results of ESN learning algorithm. Figure 15 shows the training and testing results of knee and ankle angular velocities through ESN learning algorithm. ${\dot{\theta }}_{1}$ and ${\dot{\theta }}_{2}$ mean the angular velocities for knee and ankle, which are shown by blue solid lines. Red solid lines represent real angular velocities, which are recorded by IMU. As can be seen from Figure 14, the injured lower limb swings the calf at the 5th second, and then the knee periodically stretches and flexes about at about 41 s. When the knee joint angle reaches 0 rad, the knee joint swings to its maximum position. During the swing phase, the knee joint can flex more than −1.8 rad. The angle of the ankle joint is between 1.8 and 2.2 rad, which is always plantar flexion. Figure 15 shows that the angular velocity of the knee joint alternates between −2 and 2 rad/s with a cycle of about 4 s, while the angular velocity of the ankle joint varies slightly between −0.5 and 0.5 rad/s. The motion intention of injured lower limbs can be identified by the ESN learning algorithm with multichannel sEMG signals from 32 to 45.3 s. Meanwhile, it is also inferred that the proposed method shows superior performance for intention identification of injured lower limb.

The second one is an MPC problem; the joint trajectories of injured lower limb can be identified via an ESN model based on active motion intention, which is viewed as desired trajectories of the two-link lower limb rehabilitation robot. The control law generated by the Algorithm 1 (PASHS) is transmitted to two-link lower limb rehabilitation robot, which aims at assisting patient to do rehabilitation training. The next predictive state also can be computed by the optimization results of MPC problem (2), which feeds back to the two-link lower limb rehabilitation robot system. Meanwhile, the MPC problem can be seen as follows:

(37) $$\begin{array}{l} \underset{\theta^{k},{\dot{\theta }}^{k},\tau^{k}}{\text{min}}\overset{N}{\underset{i=1}{\sum }}{\left\|\theta \left(k+i|k\right)-\theta^{\text{d}}\left(k+i|k\right)\right\|}_{Q}^{2}+\overset{N_{\tau }-1}{\underset{j=0}{\sum }}{\left\|\Delta \tau \left(k+j|k\right)\right\|}_{R}^{2}\\ \text{s.t.}\left[\begin{array}{c} {\dot{\theta }}^{k_{i}}\\ {\ddot{\theta }}^{k_{i}} \end{array}\right]=\left[\begin{array}{cc} 0 & I_{2\times 2}\\ 0 & -{\text{D}}^{-1}\left(\theta^{k_{i}}\right)\text{C}\left(\theta^{k_{i}},{\dot{\theta }}^{k_{i}}\right) \end{array}\right]\left[\begin{array}{c} \theta^{k_{i}}\\ {\dot{\theta }}^{k_{i}} \end{array}\right]+\left[\begin{array}{c} 0\\ {\text{D}}^{-1}\left(\theta^{k_{i}}\right) \end{array}\right]\tau^{k_{j}}\\ \;-\left[\begin{array}{c} 0\\ {\text{D}}^{-1}\left(\theta^{k_{i}}\right)\text{G}\left(\theta^{k_{i}}\right) \end{array}\right],\\ \;\theta \left(k+i|k\right)\in \left[\theta_{\text{min}},\theta_{\text{max}}\right],\dot{\theta }\left(k+i|k\right)\in \left[{\dot{\theta }}_{\text{min}},{\dot{\theta }}_{\text{max}}\right],\\ \tau \left(k+j|k\right)\in \left[\tau_{\text{min}},\tau_{\text{max}}\right],\\ \;i=1,2,\ldots ,N,j=1,2,\ldots ,N_{\tau }, \end{array}$$

where *k*_*i,j*_ ≜ *k* + *i, j*|*k*, and ***θ***^*k*^, ${\dot{\theta }}^{k}$, and ***τ***^*k*^ represent the angle vector, angular velocity vector, and torque vector of two-link lower limb rehabilitation robot at prediction horizon and control horizon, respectively. ***θ***^d^(*k* + *i*|*k*) means the desired trajectory at *k* + *i* time, and Δ***τ***(*k* + *j*|*k*) is the control input increment. *Q* = 5*I* and *R* = *I*, where *I* is the identity matrix and the index *N* = 3 and *N*_τ_ = 3.

**Figure 16:**
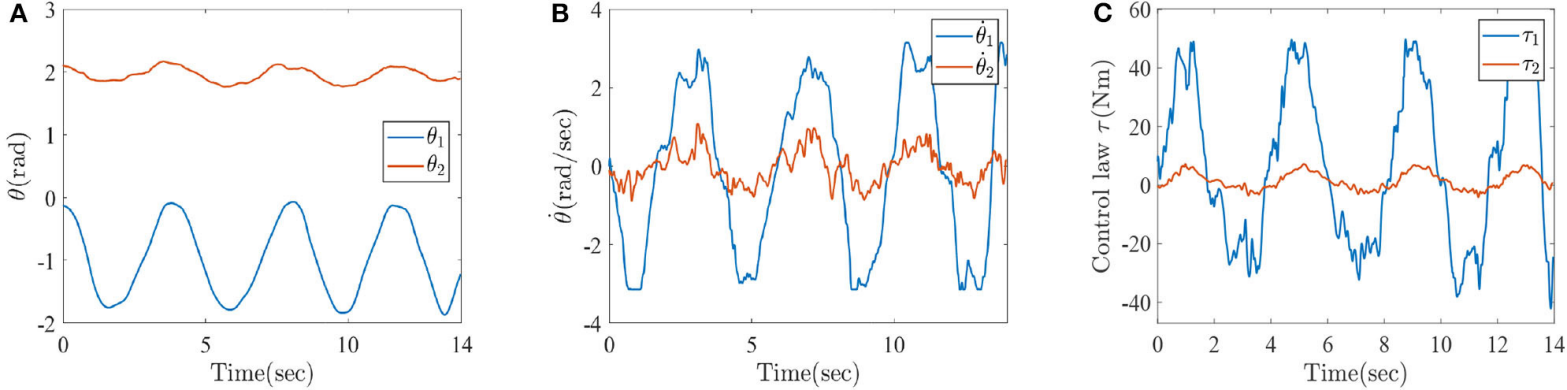
The simulation results with active rehabilitation training, **(A)** the angles of lower-limb rehabilitation robot based on active intentions, **(B)** the angular velocities of lower-limb rehabilitation robot based on active intentions, and **(C)** the torques of lower-limb rehabilitation robot.

The trained ESN model can effectively identify the joint angle and angular velocity of injured lower limb from the multichannel sEMG signals. The lower limb rehabilitation robot takes the results of recognition as the desired trajectories. Combining the ESN model and MPC technique, the human–machine interactive control method is developed, investigated, and analyzed for lower limb rehabilitation robot and injured lower limb in this paper. Besides, to design the human-machine interactive controller, the joint angle and angular velocity that can be regarded as desired trajectories are identified by ESN learning algorithm from 32 to 46 s. The numerical results are shown in Figure 16.

Figures 16A–C represent the numerical results of angles, angular velocities, and torques of lower-limb rehabilitation robot during rehabilitation training process. As shown in Figure 16, the blue solid lines mean the results of knee and the red solid lines represent the results of ankle, respectively. In light of Figure 16, it can be seen that the patients can be stably driven to do rehabilitation motion by lower limb rehabilitation robot, which be oriented by a human's active intention. It is also inferred that the rehabilitation robot appropriately generates torques, which assist patients to do rehabilitation training and avoid the second injury. It is thus further demonstrated that it is very practical to train the injured lower limb through a human–machine interactive control method with multichannel sEMG signals. In other words, it is also verified that the ESN learning algorithm and Algorithm 1 (PASHS) are feasible and effective for the rehabilitation training of injured lower limb.

## 5. Conclusions

In this paper, to obtain an optimal controller of a non-linear system, an MPC problem firstly solved by a new PASHS algorithm has been proposed and analyzed by exploiting the three-order Taylor discretization formula to linearize and discretize the constraint conditions. Furthermore, the PASHS approach not only takes advantage of a projected operator, but it also integrates the active set into HS conjugate gradient methods; the optimal controller can thus be rapidly solved for a non-linear optimization problem. Moreover, the feasibility and global convergence have been rigorously proved in this paper. Some numerical results have been presented and analyzed to substantiate the feasibility, effectiveness, and superiority of the developed human–machine interactive control method for passive/active rehabilitation training. The ESN model with multichannel sEMG signals also has been proposed for intention recognition, which could identify the joint angles and angular velocities of the injured lower limb to realize active rehabilitation training. In other words, passive rehabilitation makes patients train through fixed-based trajectories of injured lower limb; however, the desired trajectories of active rehabilitation training are identified by ESN learning algorithm with multichannel signals. Besides, combining with MPC technology and Algorithm 1 (PASHS), human-machine interactive control has been developed, investigated, and analyzed for two-link lower limb rehabilitation robot. The numerical results have inferred that the proposed method could be effectively applied to passive/active rehabilitation training. The proposed method has also solved a problem that creates uncertainty in the model. In future work, more effective and real-time methods will be developed and investigated in the solution of MPC problem and applied to the rehabilitation of patients, such as upper limb rehabilitation training, assisting patients to walk on the plane, or up and down stairs.

## Data Availability Statement

Publicly available datasets were analyzed in this study. This data can be found at: http://www.dqxy.ccut.edu.cn/2017/0906/c5519a78133/page.htm.

## Ethics Statement

Ethical review and approval was not required for the study on human participants in accordance with the local legislation and institutional requirements. Written informed consent for participation was not required for this study in accordance with the national legislation and the institutional requirements.

## Author Contributions

TS: methodology, software, and review. YT: investigation and writing original draft. ZS: conceptualization and methodology. BZ: methodology. ZP: algorithm. JY: methodology and review. XZ: editing. All authors contributed to the article and approved the submitted version.

## Conflict of Interest

The authors declare that the research was conducted in the absence of any commercial or financial relationships that could be construed as a potential conflict of interest.

## Appendix

**Table:** Symbols of the Model and Algorithm

**x**^*k*^	system state at *k*th sampling instant
**u**^*k*^	control input at *k*th sampling instant
**y**^*k*^	predicted output at *k*th sampling instant
**r**^*k*^	desired output at *k*th sampling instant
Γ	objective funtion
∇Γ	gradient of objective function
Λ, *b*	coefficient matrix and vector of equality constraint
Ω	bound constrained set
*H*(*x*) or *H*^*^	active set
*L*(*x*) or *L*^*^	free variables set
*P*_Ω_(*x*)	projection function
P	projection matrix
*d*^*k*^	search direction of algorithm
η^*k*^	step size of Armijio-type line search rule
X	echo state of ESN
U, Y	inputs and outputs of ESN
${𝔐}_{X}$, ${𝔐}_{U}$, ${𝔐}_{F}$, ${𝔐}_{Y}$,	matrixes of ESN
